# Psychomotor slowing in schizophrenia is associated with aberrant postural control

**DOI:** 10.1038/s41537-024-00534-5

**Published:** 2024-12-19

**Authors:** Melanie G. Nuoffer, Anika Schindel, Stephanie Lefebvre, Florian Wüthrich, Niluja Nadesalingam, Alexandra Kyrou, Hassen Kerkeni, Roger Kalla, Jessica Bernard, Sebastian Walther

**Affiliations:** 1 Translational Research Center, University Hospital of Psychiatry and Psychotherapy, University of Bern, Bern, Switzerland; 2 Graduate School for Health Sciences, University of Bern, Bern, Switzerland; 3 Department of Neurology, Inselspital, University Hospital Bern, University of Bern, Bern, Switzerland; 4 Department of Psychological and Brain Sciences, Texas A&M University, College Station, TX USA; 5 Texas A&M Institute for Neuroscience, Texas A&M University, College Station, TX USA; 6 Department of Psychiatry, Psychosomatics and Psychotherapy, Center of Mental Health, University Hospital of Würzburg, Würzburg, Germany

**Keywords:** Schizophrenia, Schizophrenia

## Abstract

Motor abnormalities, including psychomotor slowing, are prevalent in a large proportion of individuals with schizophrenia. While postural control deficits have been observed in this population, the impact of motor abnormalities on postural stability remains unclear. This study aimed to objectively evaluate postural stability in patients with and without psychomotor slowing and healthy controls. Seventy-three schizophrenia patients with psychomotor slowing (PS; Salpêtrière Retardation Rating Scale (SRRS) ≥ 15), 25 schizophrenia patients without psychomotor slowing (non-PS; SRRS < 15), and 27 healthy controls (HC) performed four conditions on the Kistler force plate: eyes open (EO), eyes closed (EC), head reclined with eyes open (EOHR), and head reclined with eyes closed (ECHR). Larger sway areas and higher Root Mean Square (RMS) values indicate lower postural stability, while a lower Complexity Index (CI) reflects reduced adaptability, flexibility, and dynamic functioning of postural control. PS exhibited larger sway areas and higher RMS compared to the other groups. Both PS and non-PS showed reduced complexity in postural control compared to healthy controls, without differences between the two patient groups. Reduced postural stability and complexity were associated with greater expert-rated motor abnormalities, as well as more severe negative symptoms. Additionally, lower complexity was linked to reduced physical activity levels. These findings suggest that psychomotor slowing is associated with lower postural stability, potentially reflecting impaired cerebellar function. Furthermore, the loss of complexity in postural control highlights reduced flexibility, adaptability, and efficiency in the postural control network of individuals with schizophrenia.

## Introduction

Schizophrenia is a severely disabling mental disorder that usually has its onset in adolescence^1,2^. Common features of schizophrenia are positive (e.g., hallucinations) or negative (e.g., anhedonia and social withdrawal) symptoms, cognitive impairments (e.g., poor working memory), and motor abnormalities (e.g., psychomotor slowing, catatonia, parkinsonism, and neurological soft signs (NSS))^3–6^. Motor abnormalities are associated with a more sedentary lifestyle^7^, poorer long-term psychosocial functioning^8^, and predict a more severe course of disease^9^. Abnormal psychomotor behaviour including aberrant balance and postural control are found even in antipsychotic naïve schizophrenia patients^10,11^ as well as in individuals at ultra-high risk^12,13^.

Psychomotor slowing refers to generalised hypokinesia. It affects spontaneous fine and gross motor tasks, and it is linked to poorer non-verbal social perception and gestures^14–18^. Fine motor behaviour is evaluated through writing or complex finger manipulations such as coin rotations, whereas gross motor behaviour is assessed using walking tasks, postural stability, or actigraphy to measure overall physical activity.

Neuroimaging studies linked psychomotor slowing to aberrant functional and structural connectivity between the supplementary motor area (SMA), primary motor cortex (M1), basal ganglia, thalamus, and cerebellum^19–23^. Also, it has been shown that functional connectivity at rest between the primary motor cortex, the anterior cingulate cortex and the cerebellum increases with stronger cortical inhibition in patients with schizophrenia and psychomotor slowing^24^. This may indicate that postural stability might be impaired in patients with psychomotor slowing.

One convenient property of motor abnormalities is their physical nature, making them available to instrumental quantification. Traditional expert-rating scales rely on the information disclosed by the patient or on behaviours observed in interaction, which can be confounded by suspiciousness, amotivation, and observer bias^5^. The use of sensitive instruments (e.g., Posturography System and Wii Balance Board) and relevant variables is more reliable than patients’ self-reports or visual assessment by clinicians^25,26^.

Dynamics of quiet, upright stance are complex, involving visual, vestibular, proprioceptive, and somatosensory feedback loops, multiple brain areas, and the musculoskeletal system^27^. As the cerebellum integrates and processes postural information, postural stability could be considered as an easy to implement behavioural marker of cerebellar dysfunction^28,29^. The cerebellum is not exclusively involved in motor function, but also in cognition and emotion^30^. For instance, the cerebellum has also been implicated in neurological soft signs (NSS) and negative symptoms^31–33^. Still, other brain areas such as the basal ganglia, brain stem, and motor cortex are also strongly involved in movements and should be considered when studying postural control.

Previous studies demonstrated impaired postural stability in patients with schizophrenia compared to controls^34–38^. However, they rarely considered motor abnormalities when examining postural function in patients with schizophrenia^34,35^. The most commonly chosen parameters are the sway path or the sway area, that inform on the total path length of a person’s sway and the total area that it covers^34,39,40^.

In order for an organism to function and adapt to demands of everyday life, a wide repertoire of behaviour is crucial. The integration of multiple feedback loops, control systems, and regulatory processes is a prerequisite for appropriate reactions to changing environments. This flexibility and variability of a system or organism can be quantified using a measure of complexity^41,42^, such as complexity index (CI) measured by multiscale entropy^43^. A healthy person (with a highly flexible network of neuromuscular connections) exhibits more complex dynamics in postural control and hence shows high postural control. Aging or illnesses (e.g., multiple sclerosis, scoliosis, and bipolar disorder) can compromise feedback loops, leading to reduced physiological complexity and increased swaying^41,43–46^.

This study aimed to explore cerebellar function, using postural stability markers, in schizophrenia with psychomotor slowing and to explore associations between cerebellar dysfunction and motor abnormalities. We included a natural stance condition, as well as conditions with manipulated visual (i.e., eyes closed), vestibular (i.e., head reclined), and proprioceptive (i.e., tandem stance) information. We hypothesised that patients with psychomotor slowing (PS) would show less postural control and less complexity compared to patients without psychomotor slowing (non-PS) and healthy controls (HC). We expected the deficit in postural control to increase from natural and ecological conditions (e.g., eyes open) to challenging conditions (e.g., eyes closed, head reclined, and tandem stance). In addition, we hypothesised that within patients, postural sway parameters would be associated with expert ratings of several hypokinetic motor abnormalities and negative symptoms.

## Material and methods

### Participants

We included baseline data of 98 patients fulfilling the criteria for schizophrenia spectrum disorders according to the Structured Clinical Interview for DSM-5® (SCID-5) and taking part in the recently completed prospective randomised, double-blind, placebo-controlled OCoPS-P study (Overcoming Psychomotor Slowing in Psychosis; ClinicalTrials.gov Identifier: NCT03921450)^47^. Using a cut-off score of 15 points on the Salpêtrière Retardation Rating Scale^48^ (SRRS), patients were categorised into a group with psychomotor slowing (PS, SRRS ≥ 15, *N* = 73) and without (non-PS, SRRS < 15, *N* = 25). We additionally included 27 age- and sex-matched healthy controls (HC) who neither had a history of psychiatric disorder nor any first-degree relative with schizophrenia spectrum disorder. General exclusion criteria included age <18 and >60 years, active substance abuse or dependence (except nicotine), past or current neurological disorders impacting motor behaviours, seizures, or hearing problems. All patients were recruited at the in- and out-patient departments of the University Hospital of Psychiatry and Psychotherapy in Bern, Switzerland. All except 3 patients were taking antipsychotics at the time of baseline assessments. Controls were contacted using flyers, online announcements, or word of mouth. All participants signed written informed consent prior to study participation. The study protocol adhered to the Declaration of Helsinki and was approved by the local ethics committee (2018-02164).

### Procedures and measures

#### Clinical and motor scales

We measured general psychopathology with the Positive And Negative Symptom Scale (PANSS)^49^ and negative symptoms with the Brief Negative Symptom Scale (BNSS)^50^. Psychomotor slowing was assessed using the Salpêtrière Retardation Rating Scale (SRRS)^48^. Focusing on the motor components of psychomotor slowing, the first five items plus the last one (gait, movements of limbs/trunk, movements of head/neck, verbal flow, modulation of voice, and general appreciation) were summarised in the subscore mSRRS^17,51,52^. We also assessed other motor abnormalities, such as parkinsonism (Unified Parkinson’s Disease Rating Scale—Part III, UPDRS)^53^, Catatonia (Bush-Francis Catatonia Rating Scale, BFCRS)^54^, and neurological soft signs (Neurological Evaluation Scale, NES)^55^.

To objectively measure participants’ gross movements in a day, subjects wore a tri-axial-accelerometer Move4 (movisens GmbH, Karlsruhe, Germany) continuously for 24 h on their non-dominant arm^7,21,52,56–60^. Sleeping hours (including naps) were removed before calculating the activity level per hour (AL/h) with the Movisens DataAnalyzer Software. Current antipsychotic medication was converted into olanzapine equivalents (OLZ eq.) according to Leucht^61^.

#### Measurement of postural stability

The participant’s balance was assessed using a 40 cm × 60 cm Kistler force plate (Type 9286A, Kistler Instrumente AG, Winterthur Schweiz). Participants were instructed to keep still, to relax their arms at their side, and to stand comfortably (e.g., feet approximately hip-width apart). We then manipulated visual (eyes open vs. closed), vestibular (head in natural upright position vs. head reclined), and proprioceptive inputs (feet hip-width apart vs. tandem stance). With a combination of these manipulations the origin of a deficit in postural control of patients might be identifiable.

If participants lost their balance, they could quickly hold on to a bar in front of them if necessary and/or quickly open their eyes in the eyes-closed tasks. Any occurrence of these safety behaviours (touching the bar or opening the eyes) was noted.

**Fig. 1:**
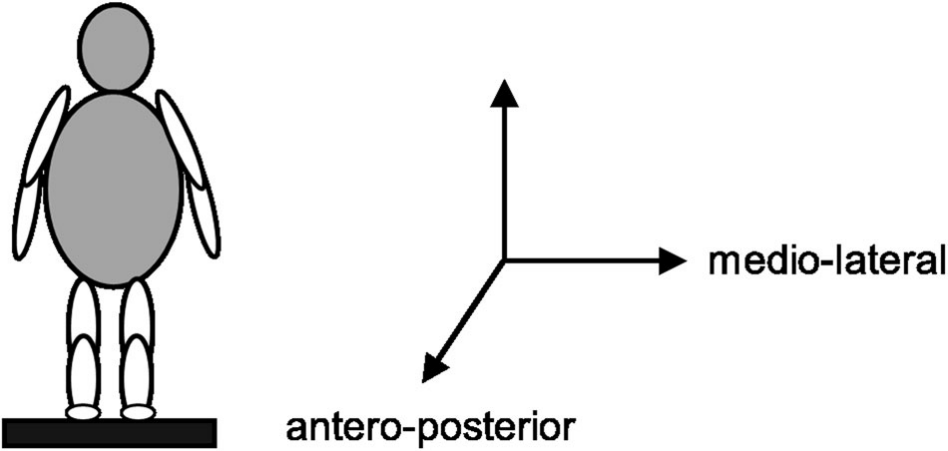
Postural sway axes. Sketch of a person standing on the Kistler platform. Arrows indicate the directions of the sway axes. Left-to-right swaying is called medio-lateral and front-to-back swaying is called antero-posterior. The third vertical arrow measures the vertical reaction force.

The centre of pressure (CoP) was recorded along the antero-posterior and medio-lateral axes (Fig. 1) with a sampling rate of 40 Hz using NeuroPlatform2^62^. Each participant performed the conditions for 30 s in the following order: (i) eyes open in a natural stance (i.e., upright head position, feet hip-width apart) (EO); (ii) eyes closed in a natural stance (EC); (iii) eyes open and head reclined (EOHR); iv) eyes closed and head reclined (ECHR); (v) eyes open and tandem stance (i.e., one foot directly in front of the other in a straight line, with toes of one foot touching the heel of the other foot) (EOTS); (vi) eyes closed and tandem stance (ECTS).

The conditions EOTS and ECTS were exceptionally difficult for all participants due to the altered proprioceptive input (tandem stance instead of feet hip-width apart). In the tandem stance, the physical basis for postural control is heavily compromised, and neither visual, vestibular, nor proprioceptive inputs can fully compensate. Hence, the disproportionally high variance in EOTS and ECTS made these conditions incomparable to the first four conditions, which is why they were analysed separately (Tables S19 and S20, Fig. S7). Sample sizes vary between conditions, as some participants were unable to correctly perform them (e.g., falling off the platform without continuous use of safety behaviours and assistance).

We used six parameters to analyse postural stability: the root mean square (RMS) of CoP displacement in medio-lateral (RMS_ml_) and antero-posterior (RMS_ap_) direction and total (RMS_total_), the sway area, and the Complexity Index (CI) in the medio-lateral (CI_ml_) and antero-posterior (CI_ap_) direction. For one participant the raw data was missing. Extreme outliers were defined as three interquartile ranges above the third or below the first quantile and removed for each parameter and each condition separately prior to analyses.

##### Root mean square

The RMS defines the displacement of all individual CoP measurements around the mean CoP for the whole measurement^63^. The RMS displays the average distance deviation from the mean of the recorded data points $\bar{({\boldsymbol{x}})}$ in mm^64^. Higher RMS indicates worse balance^65^. We used PosturographicExplorer2 (version 2.0.77.2011, 2011, Dr. Sergei Novoshilov, Dr. Siegbert Krafczyk; Formula 1 + 2)^66,67^ to convert the raw CoP data into the three RMS measures (RMS_ml_, RMS_ap_, RMS_total_). We removed 10 participants (6 PS, 2 non-PS, 2 HC) who were extreme outliers in at least one RMS measure in at least one condition. If a participant was an outlier in one RMS value, all RMS values were removed for that condition.

Formula 1 + 2.

$${{RMS}}_{{{ml}}\; {{or}}\; {{ap}}}=\sqrt{\frac{1}{N}\mathop{\sum }\limits_{i=1}^{N}{({x}_{i}-\bar{x})}^{2}}\,\,{{RMS}}_{{total}}=\sqrt{\frac{1}{N}\mathop{\sum}\limits_{i=1}^{N}{({x}_{i}-\bar{x})}^{2}+(y_{i}-\bar{\rm{y}})^{2}}$$

*Note*. *N* = total number of measurement points over time; $\,{x}_{{i}}$ = individual measured values in medio-lateral or antero-posterior direction; $\bar{x}$ = mean of the measured data points

##### Sway area

**Fig. 2:**
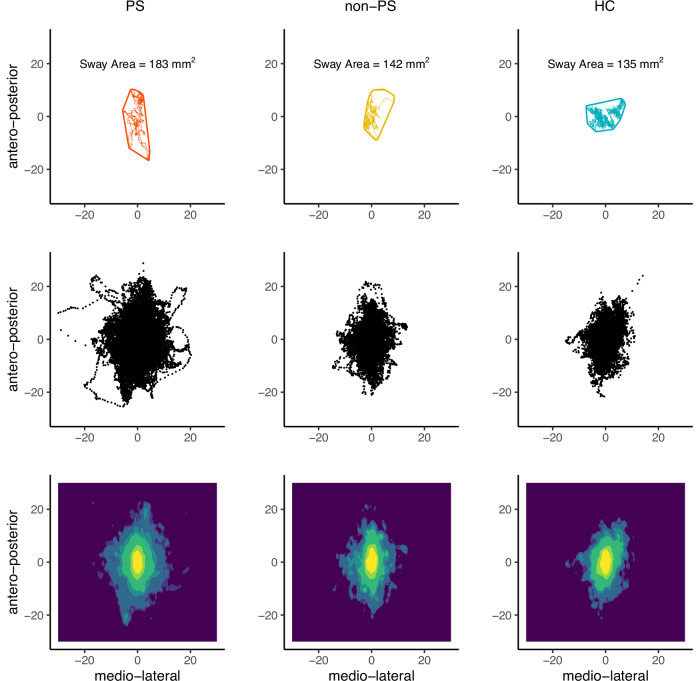
Individual sway path, group sway area, and group sway area density during EO condition. *Note*. Same x- and y-scale is used for all plots to increase comparability (mm). Other conditions are presented in the supplements (S-Figs. 1 and 2). Top: Sway path and border defining the sway area of three individuals that best represent the median of their group during EO condition. *Red* PS, *yellow* non-PS, *blue* HC. Middle: scatterplot of all CoP measuring points of all individuals of the three groups together during EO condition. Bottom: Density plot illustrating the dispersion and height of the scatterplot above. Colour scale ranges from yellow (highest density of CoP data points) to dark purple (no or almost no CoP data points).

The sway area represents the area that encloses the CoP data points (mm^2^). A large sway area results from more body swaying, i.e., worse balance^68^. The sway area was calculated from the raw CoP data in Matlab (MATLAB Version: 9.13.0.2166757 (R2022b) Update 4) using the convex hull method (Fig. 2, Figs. S1 and S2)^69^. We excluded the sway area of 12 participants (7 PS, 3 non-PS, 2 HC) as extreme outliers. Only the extreme condition was removed, while the other conditions of that participant were included.

##### Complexity index

Complexity in postural sway reflects the adaptability and flexibility of the postural control system^42,43^. Higher complexity indicates a more dynamic and responsive system, capable of making varied and timely corrections to maintain balance, it should result in less overall sway. In contrast, reduced complexity indicates a less responsive postural system, associated with more predictable and larger sway patterns (Tables S14 and S15, Fig. S5).

Sample entropy quantifies the irregularity of temporal patterns in a given signal. Multiscale sample entropy extends the traditional sample entropy by assessing the entropy of a signal at various levels of temporal resolution (i.e., coarse-graining the data to different timescales), thereby capturing both short-term and long-term dynamics^43,70^. The complexity index reflects the area under the curve of sample entropy versus timescale (*τ*)^41,43,70^. For more in-depth explanation on the calculation refer to the Supplements.

We calculated the complexity index (CI) separately for the raw medio-lateral and antero-posterior CoP data using Matlab, using *τ* = 6 timescales, a similarity threshold (*r*) of 15% and a sequence length (*m*) of 2, as recommended in the literature^43,70,71^.

The complexity index showed no outliers; however, one participant, who was an outlier in both RMS and sway area across both conditions with the head reclined, was excluded from the complexity analysis for these conditions.

### Statistical analyses

**Fig. 3:**
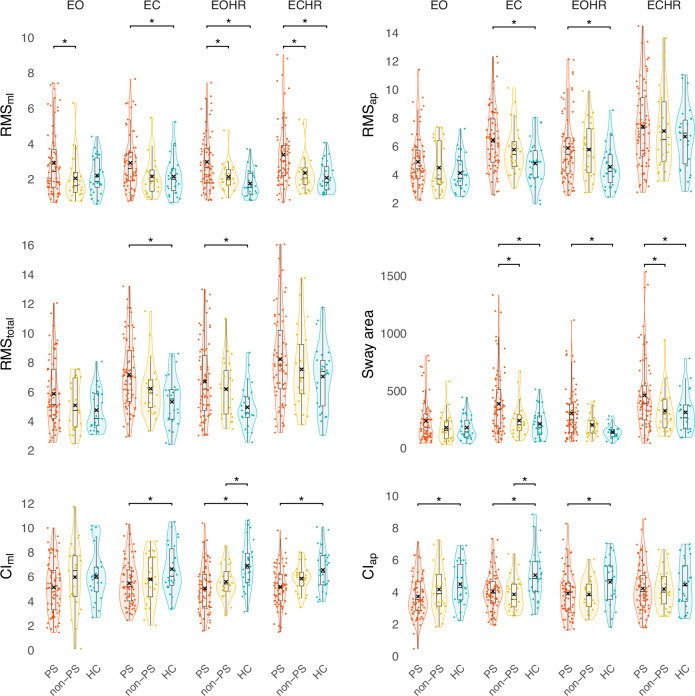
Differences between groups per condition for postural parameters. *Note*. Points show individual measurements, violin the approximate distribution, boxplot the quartiles (1st, median, 3rd), black cross indicates the mean. *Red* PS, *yellow* non-PS, *blue* HC, *EO* eyes open, natural upright head position, and feet hip-width apart, *EC* eyes closed natural upright head position, and feet hip-width apart, *EOHR* eyes open, head reclined, and feet hip-width apart, *ECHR* eyes closed, head reclined, and feet hip-width apart, *PS* patients with psychomotor slowing, *non-PS* patients without psychomotor slowing, *HC* healthy controls, *RMS*_*ml*_ Root Mean Square medio-lateral, *RMS*_*ap*_ Root Mean Square antero-posterior, *RMS*_*total*_ Root Mean Square for total deviation, *Cl*_*ml*_ Complexity Index medio-lateral, *Cl*_*ap*_ Complexity Index antero-posterior. * *p*_corr_ < 0.05.

All analyses were done using R (4.3.0) and RStudio (2023.06.0 + 421). We calculated the extreme outliers (three interquartile ranges above the third or below the first quantile) for each parameter and each condition separately. Sample sizes vary slightly between analyses. Also, some data is not available for some participants (e.g., refusal of actigraphy). A *p*-value < 0.05 was considered to be significant. We used false discovery rate (FDR) to account for multiple comparisons (*p*_corr_). Demographic and clinical characteristics were compared with Mann–Whitney *U* tests, ANOVAs, and chi-square tests (Table 1). We used a repeated-measures ANOVA for each postural parameter including 3 groups (Table 2, Fig. 3, Table S1) and 4 conditions (Table S2). *P*-values of 12 main effects and 6 interactions are corrected for multiple comparisons using FDR (*p*_corr_).

**Table 1:** Demographic and clinical characteristics.

	PS	non-PS	HC	Comparison	
	*N /* Mean ± *sd*			Test value	*p*-Value
*N* total	73	25	27	–	–
Sex (*N*/%) female	36/49%	14/56%	12/44%	*X*^2^ = 0.7	0.70
Age (years)	35.9 ± 13.0	23.3 ± 12.2	35.3 ± 12.4	*F* = 0.0	0.978
BMI	24.8 ± 5.2	25.7 ± 5.1	23.3 ± 3.7	*F* = 1.7	0.195
Education (years)	13.0 ± 2.4	12.7 ± 1.8	16.1 ± 3.0	*F* = 18.0	< 0.001*
				PS vs. HC: *p* < 0.001* PS vs. non-PS: *p* = 0.906 non-PS vs. HC: *p* < 0.001*	
Duration of illness (years)	10.0 ± 11.0	8.9 ± 12.1	–	*W* = 1027	0.352
Number of episodes	4.5 ± 4.4	6.4 ± 12.0	–	*W* = 959	0.708
OLZ eq.	15.4 ± 11.2	14.8 ± 10.2	–	*W* = 917	0.974
SRRS	23.4 ± 5.8	8.4 ± 2.8	–	*W* = 1825	< 0.001*
mSRRS	10.7 ± 3.1	2.9 ± 1.7	–	*W* = 1800	< 0.001*
UPDRS	21.5 ± 11.4	9.0 ± 6.0	–	*W* = 1545	< 0.001*
BFCRS	5.2 ± 4.0	1.3 ± 1.6	–	*W* = 1557	< 0.001*
NES total	15.8 ± 10.9^∆^	10.0 ± 5.5	–	*W* = 1155	0.018*
sensory integration	2.9 ± 2.7^∆^	1.7 ± 1.1	–	*W* = 1113	0.042*
motor coordination	2.2 ± 2.4^•^	0.76 ± 1.1	–	*W* = 1274	0.001*
sequencing	4.5 ± 3.4^∆^	3.1 ± 2.5	–	*W* = 1098	0.059
others	6.1 ± 5.1^∆^	4.5 ± 3.8	–	*W* = 1016	0.233
Activity level	11,961 ± 4599^υ^	19,480 ± 7406^†^	19,706 ± 7493	*F* = 23.5	< 0.001*
				PS vs. HC: *p* < 0.001* PS vs. non-PS: *p* < 0.001* non-PS vs. HC: *p* = 0.990	
PANSS total	78.0 ± 16.8	65.7 ± 14.0	–	*W* = 1269	0.004*
Positive	15.4 ± 5.2	16.7 ± 5.1	–	*W* = 766	0.233
Negative	23.1 ± 6.4	15.3 ± 4.0	–	*W* = 1571	< 0.001*
General	39.4 ± 9.1	33.7 ± 8.0	–	*W* = 1241	0.007*
BNSS total	42.6 ± 13.2	25.6 ± 10.2	–	*W* = 1567	< 0.001
Anhedonia	10.8 ± 4.2	6.5 ± 3.6	–	*W* = 1444	< 0.001
Distress	2.9 ± 1.7	2.0 ± 1.5	–	*W* = 1225	0.010*
Asocial	6.7 ± 2.4	5.6 ± 2.7	–	*W* = 1102	0.121
Avolition	6.9 ± 2.7	5.3 ± 2.5	–	*W* = 1243	0.007*
Affect	10.6 ± 3.8	5.0 ± 3.1	–	*W* = 1588	< 0.001*
Alogia	4.7 ± 3.2	1.2 ± 1.8	–	*W* = 1503	< 0.001*

*Note*. Sample sizes vary due to missing data: ∆*N* = 70, •*N* = 71, υ*N* = 66, †*N* = 24.

*PS* patients with psychomotor slowing, *non-PS* patients without psychomotor slowing, *HC* healthy controls, *N* number of participants, *sd* standard deviation, *BMI* body mass index, *OLZ eq.* olanzapine equivalent in mg/day, *SRRS* Salpêtrière Retardation Rating Scale, *mSRRS* motor part of the SRRS, *UPDRS* Unified Parkinson’s Disease Rating Scale part III, *BFCRS* Bush-Francis Catatonia Rating Scale, *NES* Neurological Evaluation Scale, *PANSS* Positive And Negative Syndrom Scale, *BNSS* Brief Negative Symptom Scale.

* *p* < 0.05.

**Table 2:** Effect of group and condition on postural stability for each posture parameter (pcorr).

	Group			Condition			Group*Condition		
	*F*	numDF, denDF	*p*_corr_	*F*	numDF, denDF	*p*_corr_	*F*	numDF, denDF	*p*_corr_
RMS_ml_	9.03	2122	< 0.001*	4.70	3353	0.007*	1.17	6353	0.416
RMS_ap_	4.95	2122	0.017*	48.50	3353	< 0.001*	0.95	6353	0.485
RMS_total_	6.91	2122	0.004*	43.01	3353	< 0.001*	0.68	6353	0.677
Sway area	7.50	2121	0.003*	40.67	3346	< 0.001*	1.02	6346	0.465
CI_ml_	7.91	2121	0.002*	1.10	3360	0.420	1.38	6360	0.306
CI_ap_	4.52	2121	0.023*	2.41	3360	0.109	1.93	6360	0.112

*Note*. *RMS*_*ml*_ Root Mean Square medio-lateral, *RMS*_*ap*_ Root Mean Square antero-posterior, *RMS*_*total*_ Root Mean Square for total deviation, *Cl*_*ml*_ Complexity Index medio-lateral, *Cl*_*ap*_ Complexity Index antero-posterior, *p*_corr_ FDR-corrected *p*-values.

* *p*_corr_ < 0.05.

**Fig. 4:**
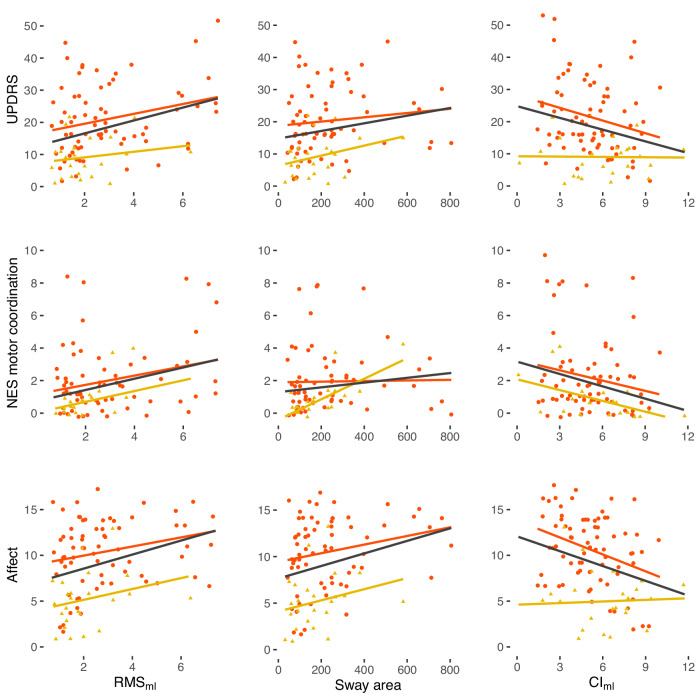
Detailed view on correlations between postural parameters and clinical rating scales across all patients. Note. Scatterplots show the association between selected postural parameters and clinical rating scales. Datapoints: *red circles* PS, *yellow triangles* non-PS. Regression lines: *red* PS, *yellow* non-PS, *black* correlation within total sample. *RMS*_*ml*_ Root Mean Square medio-lateral, *Cl*_*ml*_ Complexity Index medio-lateral, *UPDRS* Unified Parkinson’s Disease Rating Scale part III, *NES* Neurological Evaluation Scale.

Further, we correlated the six postural parameters with clinical rating scales (mSRRS, UPDRS, BFCRS, NES total and subscores, BNSS total and subscores), as well as activity level using Kendall’s Tau for the EO condition across all patients (PS and non-PS) (Table 3, Figs. 4 and S3). All *p*-values are corrected using FDR (*p*_corr_). In the Supplements we included these associations separated for PS and non-PS in EO (Table S9), the associations across patients for the other conditions (Tables S10–S12), and across all participants for activity level (Table S13, Fig. S4).

**Table 3:** Associations between postural parameters during EO and expert rated motor scores, activity level, and BNSS subscores across all patients.

	RMS_ml_			RMS_ap_			RMS_total_			Sway area			CI_ml_			CI_ap_		
	*N*	Tau	*p*_corr_	*N*	Tau	*p*_corr_	*N*	Tau	*p*_corr_	N	Tau	*p*_corr_	N	Tau	*p*_corr_	*N*	Tau	*p*_corr_
mSRRS	94	0.188	0.030*	94	0.141	0.086	94	0.173	0.040*	92	0.129	0.121	98	−0.193	0.023*	98	−0.195	0.023*
UPDRS	94	0.205	0.023*	94	0.209	0.023*	94	0.237	0.020*	92	0.174	0.040*	98	−0.178	0.031*	98	−0.167	0.040*
BFCRS	94	0.201	0.023*	94	0.089	0.261	94	0.142	0.086	92	0.112	0.173	98	−0.205	0.023*	98	−0.073	0.327
NES total	91	0.165	0.050*	91	0.214	0.023*	91	0.244	0.020*	89	0.181	0.038*	95	−0.165	0.047*	95	−0.108	0.172
NES sensory integration	91	0.182	0.041*	91	0.138	0.111	91	0.184	0.040*	89	0.211	0.023*	95	−0.105	0.196	95	0.005	0.945
NES motor coordination	92	0.225	0.023*	92	0.179	0.048*	92	0.233	0.023*	90	0.206	0.030*	96	−0.220	0.023*	96	−0.110	0.185
NES sequencing	91	0.103	0.202	91	0.170	0.050*	91	0.211	0.023*	89	0.096	0.240	95	−0.097	0.216	95	−0.040	0.588
NES others	91	0.123	0.135	91	0.185	0.035*	91	0.191	0.030*	89	0.157	0.066	95	−0.126	0.121	95	−0.129	0.089
Activity level	86	−0.143	0.086	86	−0.129	0.121	86	−0.122	0.135	85	−0.112	0.172	90	0.146	0.072	90	0.200	0.023*
BNSS total	94	0.241	0.020*	94	0.121	0.126	94	0.152	0.061	92	0.187	0.030*	98	−0.249	0.020*	98	−0.148	0.061
BNSS Anhedonia	94	0.188	0.030*	94	0.084	0.276	94	0.103	0.192	92	0.124	0.129	98	−0.203	0.023*	98	−0.079	0.290
BNSS Distress	94	0.171	0.050*	94	0.079	0.319	94	0.090	0.267	92	0.096	0.243	98	−0.207	0.023*	98	−0.119	0.147
BNSS Asocial	94	0.163	0.056	94	0.076	0.322	94	0.099	0.216	92	0.129	0.126	98	−0.155	0.061	98	−0.054	0.463
BNSS Avolition	94	0.211	0.023*	94	0.083	0.290	94	0.109	0.183	92	0.166	0.054	98	−0.202	0.023*	98	−0.058	0.438
BNSS Affect	94	0.234	0.022*	94	0.148	0.071	94	0.174	0.040*	92	0.209	0.023*	98	−0.214	0.023*	98	−0.193	0.023*
BNSS Alogia	94	0.158	0.061	94	0.057	0.456	94	0.079	0.306	92	0.153	0.070	98	−0.197	0.023*	98	−0.115	0.149

*Note*. *N* number of PS and non-PS together, *RMS*_*ml*_ Root Mean Square medio-lateral, *RMS*_*ap*_ Root Mean Square antero-posterior, *RMS*_*total*_ Root Mean Square for total deviation, *mSRRS* motor part of the SRRS, *UPDRS* Unified Parkinson’s Disease Rating Scale part III, *BFCRS* Bush-Francis Catatonia Rating Scale, *NES* Neurological Evaluation Scale, *BNSS* Brief Negative Symptom Scale. *Tau*: Kendall’s Tau correlation coefficient. *p*_corr_: *p*-values were FDR-corrected for all associations together.

* *p*_corr_ < 0.05.

Sensitivity analyses demonstrated the robustness of our findings in the light of potential confounders such as, age, sex, BMI, safety behaviours, outliers, and medication (Tables S3–S5, S16–S18, Fig. S6). Furthermore, several additional analyses such as the two-way and three-way ANOVAs with factors “vision” and “vestibular” across conditions (Tables S6–S8), associations of swaying and complexity (Tables S14 and S15, Fig. S5), analyses for the tandem stance conditions (Tables S19 and S20, Fig. S7), sway path (Table S23), and sway velocity (Table S24) are reported in the Supplements.

## Results

### Demographics and clinical data

Groups did not differ in age, BMI, or sex distribution. PS had higher PANSS total scores, more negative symptoms (e.g., BNSS total), and motor abnormalities (e.g., mSRRS, UPDRS, BFCRS, and NES total) than non-PS. Additionally, PS had lower activity levels than non-PS and controls according to actigraphy (Table 1).

### Group differences

For every postural parameter we found a main effect of group, with inferior performance of PS vs. controls. Except in CI_ml_ and Cl_ap_ there was also a main effect of condition (Fig. 3, Table 2, Table S1). In most postural parameters and conditions, non-PS presented a mean value in-between the PS and controls. No group by condition interactions were present.

#### Root mean square

Post-hoc pairwise comparisons revealed a higher RMS in PS compared to the controls when manipulating either visual or vestibular input or both. Group differences appear to be bigger in the medio-lateral than in the antero-posterior direction, as in the medio-lateral direction there are also group differences between PS and non-PS.

#### Sway area

PS show a larger sway area compared to controls when eyes are closed and/or the head is reclined. PS and non-PS solely differ when only the visual input was omitted (EC).

#### Complexity index

In CI_ml_, we noted lower complexity in PS compared to controls when the visual or vestibular system is challenged but found no difference during EO. In contrast, in PS the CI_ap_ is already reduced compared to controls in the most ecological condition (EO). Notably, only in the complexity we see differences between non-PS and controls.

### Correlations

The severity of psychomotor slowing, parkinsonism, catatonia, and NSS is associated with a higher RMS and a lower CI (Table 3, Fig. 4, Fig. S3) in EO. Parkinsonism and NSS also correlate with sway area. RMS_ml_ and CI_ml_ are consistently associated with all BNSS subscores, except Asocial. The subscore Affect shows a correlation with all postural parameters except RMS_ap_. Furthermore, more activity is associated with higher complexity in antero-posterior direction across patients; the association in ml-direction was no longer significant after FDR-correction.

### Sensitivity analyses

Results of all further analyses are in the Supplements (Tables S6–S15, S21–S24, Fig. S4, S5, and S8). Potential confounders did not change the results considerably (Tables S3–S5, S16–S18, Fig. S6).

In EOTS, groups showed similar differences as the presented conditions. ECTS was very difficult for all groups, which is visible in the sway area in ECTS that is around 8 times larger than in EOTS and around 20 times larger than in EO (Tables S19 and S20, Fig. S7).

## Discussion

This study aimed to explore cerebellar function in schizophrenia patients with psychomotor slowing (PS). We measured postural stability in schizophrenia patients with and without psychomotor slowing (PS and non-PS) and healthy controls (HC) using a Kistler platform in four conditions. In line with our first hypothesis, PS are less stable compared to controls, especially in the medio-lateral direction. Also, patients with schizophrenia (PS and non-PS) showed lower complexity in their postural stability compared to controls in both swaying directions. In line with our second hypothesis, we found associations between postural parameters and expert-rated motor abnormalities, negative symptoms, and activity level.

### Postural stability

PS are less stable compared to controls in multiple postural conditions, specifically in the medio-lateral direction. In the most natural condition EO, PS can compensate for potential deficits, which is no longer possible when visual or vestibular input is limited, leading to greater group differences in difficult conditions (EC, EOHR, and ECHR). Non-PS generally show an intermediate position.

Irrespective of motor abnormalities, patients with schizophrenia had demonstrated impaired balance compared to controls^34–36,38,72^. Our results extend previous reports, as motor abnormalities greatly influence postural sway, surpassing the impact of a schizophrenia diagnosis. Data of non-PS were closer to controls than PS. We found no interaction between groups and conditions, although non-PS seem to be closer to controls during natural tasks and closer to PS in difficult tasks. Importantly, PS were impaired in all conditions, pointing to specific dysfunctions in PS, for example in the cerebellar circuits. Postural stability is an ideal behavioural marker for the functioning of the cerebellum, as the cerebellum is strongly involved in the maintenance of balance by integrating visual, proprioceptive, and vestibular sensory inputs, fine-tuning the timing, and correcting motor responses^35,73^. There are consistent reports of cerebellar dysfunctions in schizophrenia from eye-blink conditioning^74^, finger-tapping tasks^75,76^, and neuroimaging studies^20,24,40,77–80^. Cerebellar dysfunctions are thought to contribute to the cognitive dysmetria hypothesis in schizophrenia via the cortico-cerebellar-thalamic-cortical circuit (CCTCC), leading to problems in prioritising, processing, coordinating, and appropriately responding to information^77,81,82^.

In addition to the cerebellum, other brain structures are also involved in integrating sensory, visual, or proprioceptive information for postural control^13^. For example, the basal ganglia or the vestibular system play an important role in postural control and have also been shown to be compromised in schizophrenia^36,83–86^. Therefore, future research should focus on clarifying the simultaneous involvement of multiple brain regions.

Methods in prior studies led to equivocal findings^34,72^. Here, we demonstrate that all participants sway more in antero-posterior direction than in medio-lateral direction, which is due to biomechanical properties of the ankle joint and the foot placement^87^. Interestingly, we find stronger group differences in medio-lateral direction than in antero-posterior direction, possibly due to our highly motorically impaired sample.

Our results showed that there is no difference in the postural stability of controls and PS if all sensory inputs are unrestricted (EO). There are previous studies that support our finding that balance in schizophrenia patients is impaired in the absence of visual input^34,36^, while others do not^35,72^.

Structural and functional alterations are frequently found in the motor system of patients with schizophrenia, which are associated with motor abnormalities^20,79,82,88–91^. Alterations in the motor system in schizophrenia span cerebral blood flow^22,92^, white matter integrity^21,93^, resting-state and task fMRI^24,78,90^, and effective connectivity^76^. Also, patients with schizophrenia exhibit reduced cerebellar volumes compared to controls^94,95^, and lower cerebellar grey matter density^24^. Two meta-analyses observed reduced cerebellar grey matter volumes already in first-episode patients with schizophrenia and in people at clinical high risk of psychosis^90,91^. Also, the cerebellum shows altered connectivity to the motor cortices, the thalamus, and the basal ganglia during resting-state functional connectivity^88,96–100^. Importantly, this dysconnectivity is already present in subjects at clinical high risk of psychosis^40^ and there might even be longitudinal associations between aberrant CCTCC connectivity and conversion to psychosis and/or positive symptom progression^98,101^.

We speculate that these alterations in the cerebellum could be a distinct neural substrate for postural impairments in schizophrenia and might explain the deficits we found in this study. Bernard et al. demonstrated that individuals at ultra-high risk have increased postural sway and reduced resting-state functional connectivity between the cerebellum and the cortex across multiple networks compared to controls and an association between postural sway and resting-state functional connectivity^40^. This points to a potential deficit in sensory integration already at high risk for schizophrenia and supports the cognitive dysmetria hypothesis.

In summary, there is compelling evidence of structural and functional alterations in the cerebellum and its involvement in schizophrenia and in postural deficits. Nevertheless, the implications of these findings remain largely unclear.

### Complexity

Patients with schizophrenia showed lower complexity in their postural stability compared to controls in both swaying directions. Unexpectedly, we found no effect of psychomotor slowing, although PS consistently had slightly lower complexity in medio-lateral direction than non-PS. Still, complexity in postural stability seems to be affected in schizophrenia in general and is not substantially reduced by psychomotor slowing.

Non-linear analyses such as complexity have been used in the last 30 years to investigate various data in ageing and disease^27,34,42,44^. Despite compelling and growing evidence of its relevancy, still, complexity remains an emerging field and functional implications are largely unknown^27^. The complexity in CoP dynamics in postural control is affected by ageing and disease. Neuropsychiatric disorders may affect postural control and reduce complexity^44,46^.

This is the first study to investigate complexity in postural control in schizophrenia using multiscale sample entropy as a measure of complexity. Kent et al. have investigated postural control deficits in schizophrenia using detrended fluctuation analysis, which itself is not a measure of complexity, but can be related to signal complexity^34^. Moreover, we are the first to focus on motor abnormalities in schizophrenia in the context of postural stability and complexity.

Likewise, Kent et al. found lower complexity in both medio-lateral and antero-posterior direction in patients with schizophrenia compared to controls at trend level. Furthermore, they detected a main effect of condition (eyes open vs. closed and feet together vs. shoulder-width apart) in both directions. They concluded that schizophrenia might be associated with inefficient adaptation when deprived of visual information during postural control. Instead, our complexity analysis suggests that patients with schizophrenia are inefficient in maintaining postural control regardless of the condition, as there is no main effect of condition in our data. Patients with schizophrenia show reduced complexity already in EO condition, and the complexity does not change much between conditions regardless of varying visual or vestibular input. Concurrently, PS had greater impaired postural stability measured with RMS and sway area in the complex conditions with sensory deprivation.

### Associations

In line with our second hypothesis, we found associations between postural parameters and expert-rated motor abnormalities and negative symptoms in the EO condition. Parkinsonism (UPDRS), as well as the motor coordination subscore of the NES, were most strongly associated with postural stability. The subscore Affect of the BNSS was associated with all postural parameters except RMS_ap_. Also, the CI_ml_ was associated with BNSS subscores. Interestingly, we also found a positive correlation between activity level and CI, as has previously been proposed by Busa et al. ^44^.

Negative symptoms are a key predictor of unfavourable functional outcome in patients with schizophrenia^102^. Few studies tested the association of negative symptoms with metrics of postural stability or complexity and yielded conflicting results^34,36,40,72^. We found RMS_ml_ and CI_ml_ to be associated with negative symptoms, although with a small effect.

The CCTCC might be part of the neural underpinnings for negative symptoms in schizophrenia^32,33,103^. Compromised cerebellar-(subcortical-)prefrontal connectivity was implicated with negative symptom severity in patients with schizophrenia^32,33,104^. In fact, cerebellar transcranial magnetic stimulation (TMS) rescued the cerebello-(subcortical-)cortical connectivity^33,104^, ameliorating negative symptoms in a RCT by Brady et al. ^33^, but no clinical effect was seen in another RCT by Basavaraju et al. ^104^. In sum, there is some empirical support for a causal relationship between cerebellar-(subcortical-)prefrontal dysfunctional connectivity and psychopathology which appears even in prodromal states of psychosis^40,105^.

Neurological soft signs (NSS) are minor neurological abnormalities with deficits in sensory integration, motor coordination, and sequencing of motor tasks, often associated with negative symptom severity and poor outcome^32,106,107^. We found postural deficits to be associated with stronger NSS, especially in the motor coordination subscore.

Our study is the first linking NSS with postural stability in patients with schizophrenia. Marvel et al. found no association between tardive dyskinesia and postural stability, but did not include additional motor abnormalities, such as parkinsonism and NSS^35^. Conceptually, postural stability is tied to NSS^5,108^. Difficulties in the motor coordination domain of NSS manifest as abnormalities in general coordination, balance, and gait^108^, while the NES assesses tandem walk, rapid alternating movements, finger-thumb opposition, and finger-nose pointing. Postural stability is critical to perform the tandem walk, explaining the associations we found.

Cerebellar (network) alterations might be able to explain our result of an association between NSS and postural stability. The cerebellum, SMA, and the basal ganglia form a complex integrated network, thought to contribute to NSS^24,31,109–112^. If the CCTCC-network shows aberrant activity, this could lead to aberrant motor coordination, execution, and planning^113^. Indeed, Lefebvre et al. reported an association between lower cortical inhibition in the primary motor cortex and more motor coordination deficits^24^. Following this, psychomotor slowing in psychosis might also be linked to cortical inhibitory dysfunction and more neural noise in the motor system^24^.

Our results show that there are postural stability deficits in schizophrenia, but even more so with motor abnormalities such as psychomotor slowing. Regardless of the postural condition, PS show a deficit in postural stability compared to controls. In addition, the complexity in the CoP is reduced in patients versus controls, suggesting reduced variability, flexibility, and physiological function in postural control in schizophrenia. Our results further corroborate the role of the cerebellum in abnormal psychomotor behaviour, specifically NSS, as well as in negative symptoms. Thus, NSS and negative symptoms might share neural mechanisms in schizophrenia, and postural stability might be an easily accessible screening tool and could be used for monitoring of treatment progress. Nevertheless, there are still many inconsistencies in the existing literature and a lack of research on longitudinal neural changes.

### Limitations

This study has several limitations. Firstly, we recorded daily medications but lack information on total antipsychotic exposure. However, including current medication (OLZ eq.) as a covariate in the group comparison did not affect the results. Also, no correlation has been found between antipsychotic dosage and the amount of sway^35^ and postural stability seems equally affected in unmedicated patients with schizophrenia and individuals at ultra-high risk^38–40^. Secondly, the literature is full of heterogeneity in assessing postural sway, potentially leading to inconsistent findings and hindering comparisons across studies^114^. Thirdly, no imaging data was included in this analysis to support the speculation of the cerebellum being affected, which suggest for future studies. Fourthly, this paper has placed a strong emphasis on the cerebellum due to the growing body of research linking schizophrenia, postural control, and neuroimaging findings that highlight its role. However, other brain regions, such as the basal ganglia and brainstem, also contribute to postural regulation and motor control. Future work should aim to clarify the simultaneous involvement of these regions alongside the cerebellum, offering a more comprehensive understanding of the neural mechanisms underlying postural instability in schizophrenia. Finally, postural stability and psychiatric symptoms seem to be associated cross-sectionally, while we lack information on the longitudinal development.

## Conclusion

Independent of the postural condition, patients with schizophrenia and psychomotor slowing (PS) show a deficit in postural stability and complexity compared to controls, with patients without psychomotor slowing (non-PS) in an intermediate position. Psychomotor slowing impairs postural stability, leading to differences between PS and the other groups. Complexity is reduced in schizophrenia in general, without differences between patient groups. Postural sway is related to motor abnormalities and negative symptoms, possibly due to the involvement of the cerebellum. The postural sway task might be an easily accessible clinical tool to screen and monitor treatment outcomes.

## Supplementary information

Supplementary Material

## Data Availability

Participants have not provided consent to broad data sharing of their health-related data.
