# Supplementary material for: Psychomotor slowing in schizophrenia is associated with aberrant postural control
Source: Schizophrenia (Heidelb). 2024 Dec 19;10(1):118. doi: 10.1038/s41537-024-00534-5 (PMC11659604; doi:10.1038/s41537-024-00534-5)
Supplement: Supplementary file 1 — Supplementary Material [file 41537_2024_534_MOESM1_ESM.docx]

**Supplementary Material**

**Psychomotor slowing in schizophrenia is associated with aberrant postural control**

**Authors**

Melanie G. Nuoffer^*,1,3,^
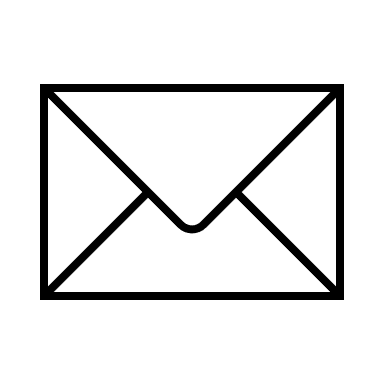
, Anika Schindel^*,1^, Stephanie Lefebvre^1^, Florian Wüthrich^1,3^, Niluja Nadesalingam^1^, Alexandra Kyrou^1^, Hassen Kerkeni^2^, Roger Kalla^2^, Jessica Bernard^4,5^, and Sebastian Walther^1,6^

**Affiliations**
1 Translational Research Center, University Hospital of Psychiatry and Psychotherapy, University of Bern, Switzerland;
2 Department of Neurology, Inselspital, University Hospital Bern, University of Bern, Bern, Switzerland;
3 Graduate School for Health Science, University of Bern, Switzerland;
4 Department of Psychological and Brain Sciences, Texas A&M University, USA

5 Texas A&M Institute for Neuroscience, Texas A&M University, USA
6 Department of Psychiatry, Psychosomatics and Psychotherapy, Center of Mental Health, University Hospital of Würzburg, Würzburg, Germany

* These authors contributed equally to this work


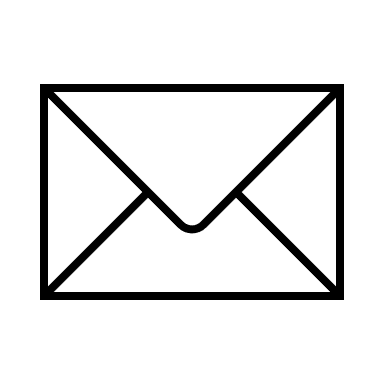
 Corresponding author
Melanie Nuoffer
Translational Research Center, University Hospital of Psychiatry and Psychotherapy, University of Bern
Bolligenstrasse 111, 3000 Bern 60
melanie.nuoffer@unibe.ch
Tel +41 78 920 68 56, Fax +41 31 632 8950

**Content**

[A) Scatterplots per group for all 4 Conditions 3](#_Toc178936685)

[B) Densityplots per group for all 4 Conditions 4](#_Toc178936686)

[C) ANOVA and Posthocs for postural parameters between groups 5](#_Toc178936687)

[D) ANOVA and Posthocs for postural parameters between conditions 6](#_Toc178936688)

[E) ANOVA and Posthocs for postural parameters between groups controlling for covariates 8](#_Toc178936689)

[a. ANOVA and Posthocs for postural parameters between groups controlling for age, sex, and BMI 9](#_Toc178936690)

[b. ANOVA and Posthocs for postural parameters between groups excluding safety behaviours 10](#_Toc178936691)

[F) ANOVA and Posthocs for postural parameters within patients with and without controlling for Medication 11](#_Toc178936692)

[G) ANOVA and Posthocs for postural parameters including factors “vision” and “vestibular” 12](#_Toc178936693)

[H) Point Plot for associations of postural parameters with motor scales, activity level, and BNSS for condition EO 15](#_Toc178936694)

[I) Associations of postural parameters with motor scales, activity level, and BNSS for EO conditions separated into PS and non-PS 16](#_Toc178936695)

[J) Associations of postural parameters with motor scales, activity level, and BNSS for conditions EC, EOHR, and ECHR 17](#_Toc178936696)

[K) Associations between activity level and postural parameters across all participants 20](#_Toc178936697)

[L) Associations between sway parameters and complexity 22](#_Toc178936698)

[M) Analyses including outliers 24](#_Toc178936699)

[N) Analyses for additional postural conditions EOTS and ECTS 29](#_Toc178936700)

[a. Frequency of Safety Behaviours 29](#_Toc178936701)

[b. Scatterplot and density plot per group for EOTS and ECTS. 30](#_Toc178936702)

[c. ANOVA and Posthocs for EOTS and ECTS separately between groups 31](#_Toc178936703)

[O) ANOVA and Posthocs for Sample Entropy over several timescales between groups 32](#_Toc178936704)

[P) ANOVA and Posthocs in postural parameters in Sway Path 35](#_Toc178936705)

[Q) ANOVA and Posthocs in postural parameters in Sway Velocity 36](#_Toc178936706)

[R) Abbreviations 37](#_Toc178936707)

# Scatterplots per group for all 4 Conditions

Figure S1. Scatterplot for EO, EC, EOHR, and ECHR per Group

PS non-PS HC


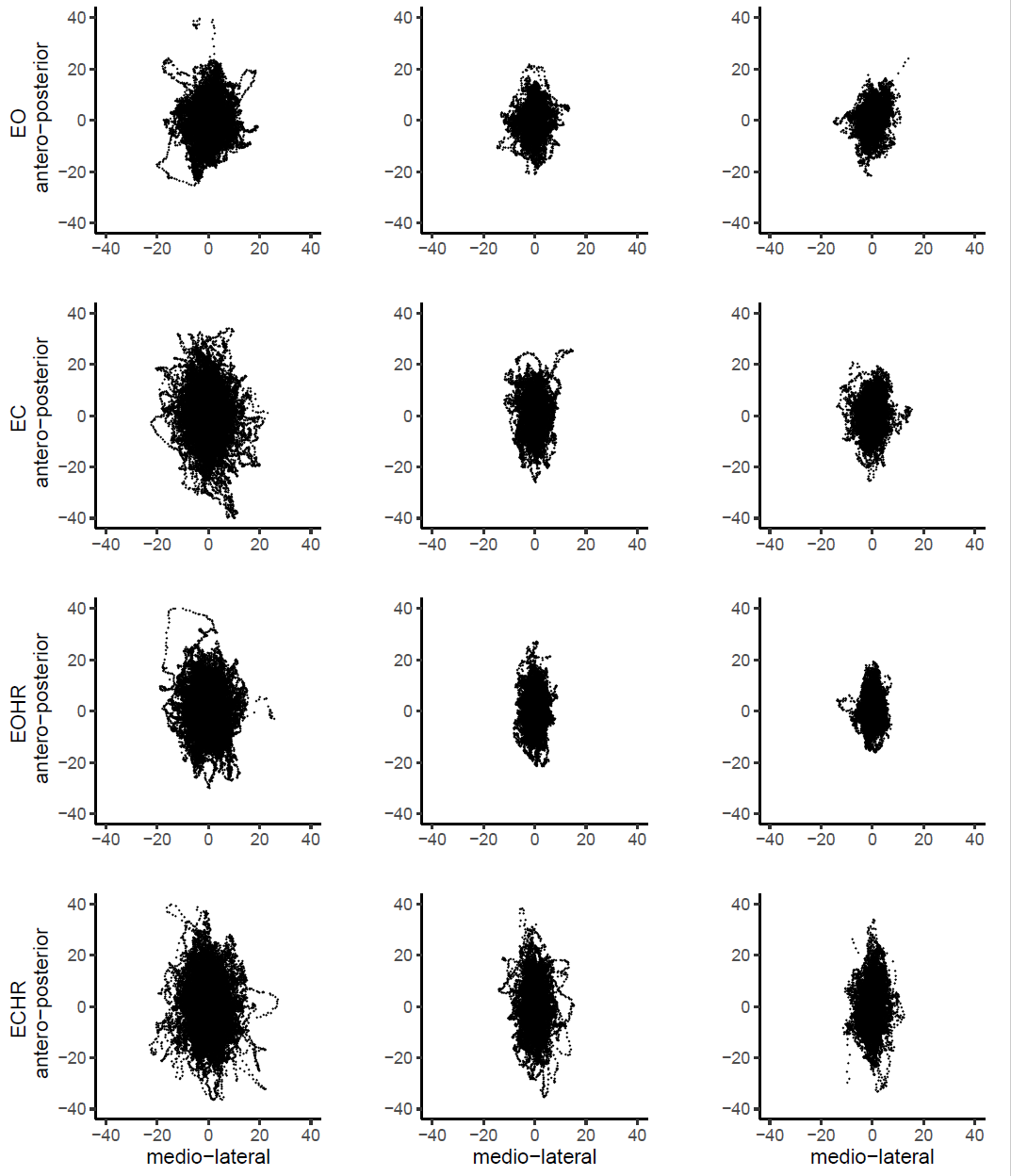


*Note.* Same x- and y-scale is used for all plots to increase comparability. Scatterplot of all CoP measuring points of all individuals of the three groups for each condition (EO, EC, EOHR, ECHR).

EO = eyes open, natural upright head position, and feet hip-width apart; EC = eyes closed natural upright head position, and feet hip-width apart; EOHR = eyes open, head reclined, and feet hip-width apart; ECHR = eyes closed, head reclined, and feet hip-width apart.

# Densityplots per group for all 4 Conditions

Figure S2. Density plot for EO, EC, EOHR, and ECHR per Group

PS non-PS HC


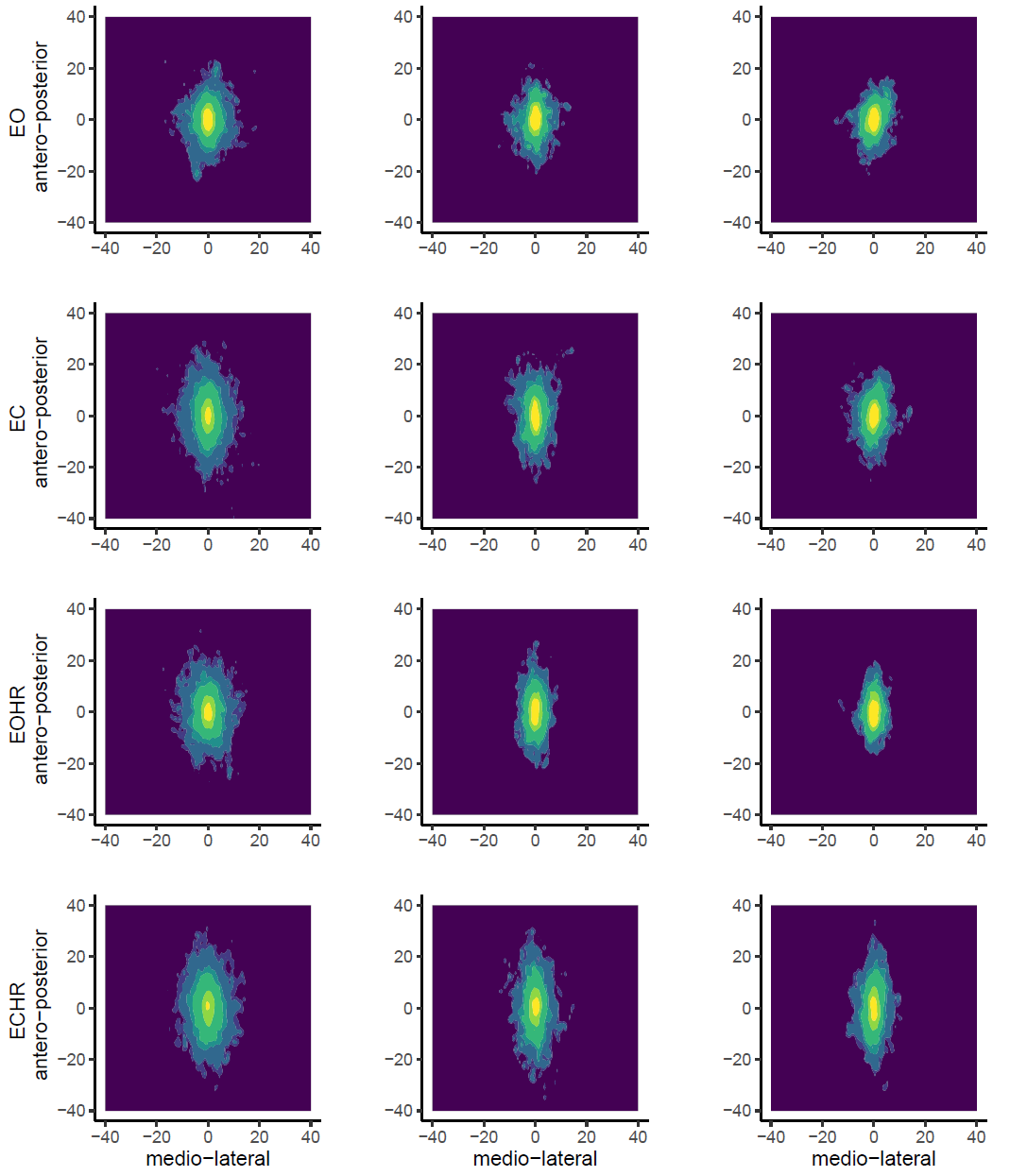


*Note*. Same x- and y-scale is used for all plots to increase comparability. Density plots illustrating the dispersion and height of the scatterplots above for each condition (EO, EC, EOHR, ECHR). Color scale ranges from yellow (highest density of CoP data points) to dark purple (no or almost no CoP data points).

EO = eyes open, natural upright head position, and feet hip-width apart; EC = eyes closed natural upright head position, and feet hip-width apart; EOHR = eyes open, head reclined, and feet hip-width apart; ECHR = eyes closed, head reclined, and feet hip-width apart.

# ANOVA and Posthocs for postural parameters between groups

The methods and results are described in the main article.

Table S1. Effect of group and condition on postural stability for each posture parameter and post-hocs for groups

|  | Main ANOVAs | | | |  |  | PS |  | non-PS |  | HC |  | p-value for posthocs btw groups | | |
| --- | --- | --- | --- | --- | --- | --- | --- | --- | --- | --- | --- | --- | --- | --- | --- |
|  |  | F | numDF, denDF | p | Conditions |  | mean ± sd | N | mean ± sd | N | mean ± sd | N | PS vs. HC | PS vs. non-PS | non-PS  vs HC |
| RMS_ml_ | Group  Condition Group*Condition | 9.03  4.70  1.17 | 2,122  3,353  6,353 | < .001*  .003*  .323 |  | EO  EC  EOHR  ECHR | 2.92 ± 1.88  2.92 ± 1.55  2.98 ± 1.55  3.38 ± 1.80 | 70  70  72  71 | 2.04 ± 1.27  2.16 ± 1.12  2.13 ± 0.75  2.35 ± 1.05 | 24  24  2525 | 2.20 ± 1.02  2.15 ± 1.08  1.75 ± 0.76  2.08 ± 0.83 | 27  27  26  26 | .090  .032*  .001*  <.001* | .036*  .056  .028*  .005* | .922  .993  .677  .868 |
| RMS_ap_ | Group  Condition Group*Condition | 4.95  48.50  0.95 | 2,122  3,353  6,353 | .009*  <.001*  .458 |  | EO  EC  EOHR  ECHR | 4.91 ± 1.85  6.45 ± 2.14  5.88 ± 2.35  7.39 ± 2.80 | 70  70  72  71 | 4.50 ± 1.69  5.77 ± 1.73  5.77 ± 1.87  7.07 ± 2.63 | 24  24  25  25 | 4.12 ± 1.23  4.79 ± 1.55  4.56 ± 1.46  6.69 ± 2.20 | 27  27  26  26 | .245  .002*  .029*  .352 | .745  .365  .968  .793 | .771  .196  .140  .825 |
| RMS_total_ | Group  Condition Group*Condition | 6.91  43.01  0.68 | 2,122  3,353  6,353 | .001*  <.001*  .677 |  | EO  EC  EOHR  ECHR | 5.87 ± 2.27  7.17 ± 2.37  6.70 ± 2.559  8.22 ± 3.06 | 70  70  72  71 | 5.08 ± 1.74  6.21 ± 1.88  6.19 ± 1.90  7.53 ± 2.62 | 24  24  25  25 | 4.75 ± 1.35  5.32 ± 1.68  4.95 ± 1.44  7.04 ± 2.23 | 27  27  26  26 | .093  .001*  .005*  .074 | .368  .177  .576  .356 | .840  .303  .184  .790 |
| Sway Area | Group  Condition Group*Condition | 7.50  40.67  1.02 | 2,121  3,346  6,346 | <.001*  <.001*  .414 |  | EO  EC  EOHR  ECHR | 235.30 ± 185.21  384.73 ± 284.49  302.08 ± 217.11  457.93 ± 320.79 | 68  72  72  69 | 177.29 ± 126.11  239.02 ± 144.90  200.22 ± 92.67  324.97 ± 186.30 | 24  24  24  25 | 177.77 ± 101.58  209.64 ± 124.06  138.65 ± 55.50  310.25 ± 173.49 | 26  26  24  25 | .216  .002*  .010*  .007* | .247  .025*  .091  .012* | .999  .794  .778  .991 |
| CI_ml_ | Group  Condition Group*Condition | 7.91  1.10  1.38 | 2,121  3,360  6,360 | <.001*  .350  .221 |  | EO  EC  EOHR  ECHR | 5.14 ± 2.10  5.47 ± 1.80  5.05 ± 1.84  5.18 ± 1.63 | 73  73  72  71 | 5.97 ± 2.70  5.79 ± 1.92  5.59 ± 1.32  5.85 ± 1.14 | 25  25  25  25 | 5.05 ± 2.19  6.62 ± 1.99  6.91 ± 1.92  6.55 ± 1.70 | 26  26  26  26 | .091  .022*  <.001*  .004* | .143  .741  .406  .232 | .987  .256  .036*  .380 |
| CI_ap_ | Group  Condition Group*Condition | 4.52  2.41  1.93 | 2,121  3,360  6,360 | .013*  .067  .075 |  | EO  EC  EOHR  ECHR | 3.72 ± 1.27  4.04 ± 1.19  3.90 ± 1.41  4.22 ± 1.46 | 73  73  72  71 | 4.16 ± 1.43  3.85 ± 1.00  3.85 ± 0.98  4.18 ± 1.18 | 25  25  25  25 | 4.49 ± 1.31  5.05 ± 1.56  4.63 ± 1.44  4.46 ± 1.49 | 26  26  26  26 | 033*  .003*  .048*  .721 | .334  .812  .981  .989 | .646  .005*  .093  .735 |

*Note*. RMS_ml_ = Root Mean Square medio-lateral; RMS_ap_ = Root Mean Square antero-posterior; RMS_total_ = Root Mean Square for total deviation; Cl_ml_ = Complexity Index medio-lateral; Cl_ap_ = Complexity Index antero-posterior; PS = patients with psychomotor slowing; non-PS = patients without psychomotor slowing; HC = healthy controls; EO = eyes open, natural upright head position, and feet hip-width apart; EC = eyes closed natural upright head position, and feet hip-width apart; EOHR = eyes open, head reclined, and feet hip-width apart; ECHR = eyes closed, head reclined, and feet hip-width apart; N = Number of participants for the specific group and condition; sd = standard deviation; btw = between.

* p < 0.05

# ANOVA and Posthocs for postural parameters between conditions

**Methods**

Methods are described in the main article.

**Results**

The condition has a significant main effect on RMS and sway area, but not on CI (Table S2). For RMSml, we exclusively see differences in PS, where only the most challenging condition ECHR showed differences with all other conditions (EO, EC, EOHR). In RMS_ap_, RMS_total_ and sway area, the majority of the conditions differ. We see more differences in PS (almost every condition except EC vs. EOHR or EO vs. EOHR) than in HC (only ECHR vs. EO, EC, EOHR). Non-PS show a pattern in-between PS and HC. There are no differences between conditions in CI, except for CI_ap_ in the PS group between EO and ECHR.

Table S2. Effect of group and condition on postural stability for each posture parameter and post-hocs for conditions

|  | Main ANOVAs | | | |  |  | EO |  | EC |  | EOHR |  | ECHR |  | p-value for posthocs between conditions | | | | | |
| --- | --- | --- | --- | --- | --- | --- | --- | --- | --- | --- | --- | --- | --- | --- | --- | --- | --- | --- | --- | --- |
|  |  | F | numDF, denDF | p | Groups |  | mean ± sd | N | mean ± sd | N | mean ± sd | N | mean ± sd | N | EO vs. EC | EO vs. EOHR | EO vs. ECHR | EC vs. EOHR | EC vs. ECHR | EOHR vs. ECHR |
| RMS_ml_ | Group  Condition Group*Condition | 9.03  4.70  1.17 | 2,122  3,353  6,353 | < .001*  .003*  .323 |  | PS  non-PS  HC | 2.92±1.88  2.04 ± 1.27  2.20 ± 1.02 | 70  24  27 | 2.92 ± 1.55  2.16 ± 1.12  2.15 ± 1.08 | 70  24  27 | 2.98 ± 1.55  2.13 ± 0.75  1.75 ± 0.76 | 72  25  26 | 3.38 ± 1.80  2.35 ± 1.05  2.08 ± 0.83 | 71  25  26 | .936  .937  .996 | .880  .986  .317 | .002*  .635  .993 | .999  .995  .446 | .014*  .933  1.000 | .019*  .826  .481 |
| RMS_ap_ | Group  Condition Group*Condition | 4.95  48.50  0.95 | 2,122  3,353  6,353 | .009*  <.001*  .458 |  | PS  non-PS  HC | 4.91 ± 1.85  4.50 ± 1.69  4.12 ± 1.23 | 70  24  27 | 6.45 ± 2.14  5.77 ± 1.73  4.79 ± 1.55 | 70  24  27 | 5.88 ± 2.35  5.77 ± 1.87  4.56 ± 1.46 | 72  25  26 | 7.39 ± 2.80  7.07 ± 2.63  6.69 ± 2.20 | 71  25  26 | <.001*  .036*  .443 | .002*  .045*  .669 | <.001*  <.001*  <.001* | .121  .999  .986 | .009*  .044*  <.001* | <.001*  .029*  <.001* |
| RMS_total_ | Group  Condition Group*Condition | 6.91  43.01  0.68 | 2,122  3,353  6,353 | .001*  <.001*  .677 |  | PS  non-PS  HC | 5.87 ± 2.27  5.08 ± 1.74  4.75 ± 1.35 | 70  24  27 | 7.17 ± 2.37  6.21 ± 1.88  5.32 ± 1.68 | 70  24  27 | 6.70 ± 2.559  6.19 ± 1.90  4.95 ± 1.44 | 72  25  26 | 8.22 ± 3.06  7.53 ± 2.62  7.04 ± 2.23 | 71  25  26 | <.001*  .078  .593 | .011*  .116  .925 | <.001*  <.001*  <.001* | .233  .997  .927 | .003*  .050*  <.001* | <.001*  .026*  <.001* |
| Sway Area | Group  Condition Group*Condition | 7.50  40.67  1.02 | 2,121  3,346  6,346 | <.001*  <.001*  .414 |  | PS  non-PS  HC | 235.30 ± 185.21  177.29 ± 126.11  177.77 ± 101.58 | 68  24  26 | 384.73 ± 284.49  239.02 ± 144.90  209.64 ± 124.06 | 72  24  26 | 302.08 ± 217.11  200.22 ± 92.67  138.65 ± 55.50 | 72  24  24 | 457.93 ± 320.79  324.97 ± 186.30  310.25 ± 173.49 | 69  25  25 | <.001*  .301  .842 | .214  .964  .948 | <.001*  .002*  .002* | .004*  .584  .531 | .001*  .244  .033* | <.001*  .010*  <.001* |
| CI_ml_ | Group  Condition Group*Condition | 7.91  1.10  1.38 | 2,121  3,360  6,360 | <.001*  .350  .221 |  | PS  non-PS  HC | 5.14 ± 2.10  5.97 ± 2.70  5.05 ± 2.19 | 73  25  26 | 5.47 ± 1.80  5.79 ± 1.92  6.62 ± 1.99 | 73  25  26 | 5.05 ± 1.84  5.59 ± 1.32  6.91 ± 1.92 | 72  25  26 | 5.18 ± 1.63  5.85 ± 1.14  6.55 ± 1.70 | 71  25  26 | .431  .963  .387 | .956  .736  .083 | 1.000  .989  .503 | .183  .949  .859 | .425  .998  .998 | .962  .893  .760 |
| CI_ap_ | Group  Condition Group*Condition | 4.52  2.41  1.93 | 2,121  3,360  6,360 | .013*  .067  .075 |  | PS  non-PS  HC | 3.72 ± 1.27  4.16 ± 1.43  4.49 ± 1.31 | 73  25  26 | 4.04 ± 1.19  3.85 ± 1.00  5.05 ± 1.56 | 73  25  26 | 3.90 ± 1.41  3.85 ± 0.98  4.63 ± 1.44 | 72  25  26 | 4.22 ± 1.46  4.18 ± 1.18  4.46 ± 1.49 | 71  25  26 | .205  .681  .166 | .670  .679  .955 | .013*  .999  .999 | .845  1.000  .410 | .677  .634  .129 | .216  .632  .919 |

*Note*. RMS_ml_ = Root Mean Square medio-lateral; RMS_ap_ = Root Mean Square antero-posterior; RMS_total_ = Root Mean Square for total deviation; Cl_ml_ = Complexity Index medio-lateral; Cl_ap_ = Complexity Index antero-posterior; PS = patients with psychomotor slowing; non-PS = patients without psychomotor slowing; HC = healthy controls; EO = eyes open, natural upright head position, and feet hip-width apart; EC = eyes closed natural upright head position, and feet hip-width apart; EOHR = eyes open, head reclined, and feet hip-width apart; ECHR = eyes closed, head reclined, and feet hip-width apart; N = Number of participants for the specific group and condition; sd = standard deviation.

* p < 0.05

# ANOVA and Posthocs for postural parameters between groups controlling for covariates

**Methods**

To ensure the robustness of our analyses to potential confounders, analyses were repeated with controlling variable covariates (age, sex, BMI, Table S3). For EC and ECHR conditions, we repeated the analyses while excluding participants who used safety behaviours (Table S4).

**Results**

Adding covariates (age, sex, BMI; safety behaviours) seemed to have no effect on our results.

Age only has a main effect on RMS_ml_, which would not survive multiple comparison correction. Also, the posthocs remain mostly identical.

Excluding the participants that used safety behaviours reduced the N by a few participants. The removal of these participants seems to improve the CI the most, as there the p-values slightly decrease. There newly is a significant main effect of condition in CI_ap_, that would however not survive FDR-correction. Posthocs remain almost identical.

## ANOVA and Posthocs for postural parameters between groups controlling for age, sex, and BMI

Table S3. Effect of group and condition on postural stability for each posture parameter controlling for age, sex, and BMI

|  | Main ANOVAs | | | |  |  | PS |  | non-PS |  | HC |  |  | p-value for posthocs btw groups | | |
| --- | --- | --- | --- | --- | --- | --- | --- | --- | --- | --- | --- | --- | --- | --- | --- | --- |
|  |  | F | numDF, denDF | p | Conditions |  | mean ± sd | N | mean ± sd | N | mean ± sd | N |  | PS vs. HC | PS vs. non-PS | non-PS  vs. HC |
| RMS_ml_ | Group  Condition  Age  Sex  BMI Group*Condition | 9.14  4.71  4.04  0.05  0.99  1.17 | 2,119  3,353  1,119  1,119  1,119  6,353 | <.001*  .003*  .047*  .818  .324  .323 |  | EO  EC  EOHR  ECHR | 2.92 ± 1.88  2.92 ± 1.55  2.98 ± 1.55  3.38 ± 1.80 | 70  70  72  71 | 2.04 ± 1.27  2.16 ± 1.12  2.13 ± 0.75  2.35 ± 1.05 | 24  24  2525 | 2.20 ± 1.02  2.15 ± 1.08  1.75 ± 0.76  2.08 ± 0.83 | 27  27  26  26 |  | .069  .023*  <.001*  <.001* | .047*  .069  .037*  .006* | .980  .952  .561  .773 |
| RMS_ap_ | Group  Condition  Age  Sex  BMI Group*Condition | 5.02  48.50  1.89  1.34  1.41  0.96 | 2,119  3,353  1,119  1,119  1,119  6,353 | .008*  <.001*  .171  .249  .237  .456 |  | EO  EC  EOHR  ECHR | 4.91 ± 1.85  6.45 ± 2.14  5.88 ± 2.35  7.39 ± 2.80 | 70  70  72  71 | 4.50 ± 1.69  5.77 ± 1.73  5.77 ± 1.87  7.07 ± 2.63 | 24  24  25  25 | 4.12 ± 1.23  4.79 ± 1.55  4.56 ± 1.46  6.69 ± 2.20 | 27  27  26  26 |  | .281  .002*  .035*  .388 | .748  .350  .965  .782 | .807  .230  .164  .862 |
| RMS_total_ | Group  Condition  Age  Sex  BMI Group*Condition | 6.98  43.05  3.19  0.87  0.45  0.69 | 2,119  3,353  1,119  1,119  1,119  6,353 | .001*  <.001*  .077  .353  .503  .662 |  | EO  EC  EOHR  ECHR | 5.87 ± 2.27  7.17 ± 2.37  6.70 ± 2.559  8.22 ± 3.06 | 70  70  72  71 | 5.08 ± 1.74  6.21 ± 1.88  6.19 ± 1.90  7.53 ± 2.62 | 24  24  25  25 | 4.75 ± 1.35  5.32 ± 1.68  4.95 ± 1.44  7.04 ± 2.23 | 27  27  26  26 |  | .103  .001*  .006*  .080 | .384  .177  .585  .360 | .844  .319  .191  .802 |
| Sway Area | Group  Condition  Age  Sex  BMI Group*Condition | 7.49  40.78  2.28  0.73  0.72  1.01 | 2,118  3,346  1,118  1,118  1,118  6,346 | <.001*  <.001*  .134  .393  .399  .418 |  | EO  EC  EOHR  ECHR | 235.30 ± 185.21  384.73 ± 284.49  302.08 ± 217.11  457.93 ± 320.79 | 68  72  72  69 | 177.29 ± 126.11  239.02 ± 144.90  200.22 ± 92.67  324.97 ± 186.30 | 24  24  24  25 | 177.77 ± 101.58  209.64 ± 124.06  138.65 ± 55.50  310.25 ± 173.49 | 26  26  24  25 |  | .160  .001*  .007*  .005* | .295  .034*  .118  .016* | .964  .667  .647  .935 |
| CI_ml_ | Group  Condition  Age  Sex  BMI Group*Condition | 8.02  1.10  3.26  0.02  1.43  1.39 | 2,118  3,360  1,118  1,118  1,118  6,360 | <.001*  .350  .074  .891  .234  .219 |  | EO  EC  EOHR  ECHR | 5.14 ± 2.10  5.47 ± 1.80  5.05 ± 1.84  5.18 ± 1.63 | 73  73  72  71 | 5.97 ± 2.70  5.79 ± 1.92  5.59 ± 1.32  5.85 ± 1.14 | 25  25  25  25 | 5.05 ± 2.19  6.62 ± 1.99  6.91 ± 1.92  6.55 ± 1.70 | 26  26  26  26 |  | .123  .032*  <.001*  .006* | .127  .706  .371  .206 | 1.00  .336  .056  .476 |
| CI_ap_ | Group  Condition  Age  Sex  BMI Group*Condition | 4.48  2.41  0.00  1.72  0.02  1.93 | 2,118  3,360  1,118  1,118  1,118  6,360 | .013*  .067  .945  .193  .879  .075 |  | EO  EC  EOHR  ECHR | 3.72 ± 1.27  4.04 ± 1.19  3.90 ± 1.41  4.22 ± 1.46 | 73  73  72  71 | 4.16 ± 1.43  3.85 ± 1.00  3.85 ± 0.98  4.18 ± 1.18 | 25  25  25  25 | 4.49 ± 1.31  5.05 ± 1.56  4.63 ± 1.44  4.46 ± 1.49 | 26  26  26  26 |  | .030*  .003*  .043*  .676 | .371  .779  .968  .979 | .586  .004*  .079  .676 |

*Note.* Posthocs are averaged over the levels of sex.

RMS_ml_ = Root Mean Square medio-lateral; RMS_ap_ = Root Mean Square antero-posterior; RMS_total_ = Root Mean Square for total deviation; Cl_ml_ = Complexity Index medio-lateral; Cl_ap_ = Complexity Index antero-posterior; PS = patients with psychomotor slowing; non-PS = patients without psychomotor slowing; HC = healthy controls; EO = eyes open, natural upright head position, and feet hip-width apart; EC = eyes closed natural upright head position, and feet hip-width apart; EOHR = eyes open, head reclined, and feet hip-width apart; ECHR = eyes closed, head reclined, and feet hip-width apart; N = Number of participants for the specific group and condition; sd = standard deviation; btw = between.

* p < 0.05

## ANOVA and Posthocs for postural parameters between groups excluding safety behaviours

Table S4. Effect of group and condition on postural stability for each posture parameter excluding participants that used safety behaviours

|  | Main ANOVAs | | | |  |  | PS |  | non-PS |  | HC |  | p-value for posthocs btw groups | | |
| --- | --- | --- | --- | --- | --- | --- | --- | --- | --- | --- | --- | --- | --- | --- | --- |
|  |  | F | numDF, denDF | p | Conditions |  | mean ± sd | N | mean ± sd | N | mean ± sd | N | PS vs. HC | PS vs. non-PS | non-PS  vs HC |
| RMS_ml_ | Group  Condition Group*Condition | 8.95  4.46  1.11 | 2,122  3,349  6,349 | <.001*  .004*  .355 |  | EO  EC  EOHR  ECHR | 2.92 ± 1.88  2.82 ± 1.45  2.98 ± 1.55  3.38 ± 1.82 | 70  68  72  69 | 2.04 ± 1.27  2.16 ± 1.12  2.13 ± 0.75  2.35 ± 1.05 | 24  24  2525 | 2.20 ± 1.02  2.15 ± 1.08  1.75 ± 0.76  2.08 ± 0.83 | 27  27  26  26 | .084  .034*  .001*  <.001* | .033*  .059  .030*  .005* | .921  .993  .675  .867 |
| RMS_ap_ | Group  Condition Group*Condition | 4.62  46.44  1.00 | 2,122  3,349  6,349 | .012*  <.001*  .423 |  | EO  EC  EOHR  ECHR | 4.91 ± 1.85  6.42 ± 2.14  5.88 ± 2.35  7.31 ± 2.74 | 70  68  72  69 | 4.50 ± 1.69  5.77 ± 1.73  5.77 ± 1.87  7.07 ± 2.63 | 24  24  25  25 | 4.12 ± 1.23  4.79 ± 1.55  4.56 ± 1.46  6.69 ± 2.20 | 27  27  26  26 | .237  .002*  .029*  .446 | .738  .384  .972  .881 | .769  .192  .137  .823 |
| RMS_total_ | Group  Condition Group*Condition | 6.55  41.0  0.70 | 2,122  3,349  6,349 | .002*  <.001*  .648 |  | EO  EC  EOHR  ECHR | 5.87 ± 2.27  7.10 ± 2.34  6.70 ± 2.55  8.16 ± 3.03 | 70  68  72  69 | 5.08 ± 1.74  6.21 ± 1.88  6.19 ± 1.90  7.53 ± 2.62 | 24  24  25  25 | 4.75 ± 1.35  5.32 ± 1.68  4.95 ± 1.44  7.04 ± 2.23 | 27  27  26  26 | .088  .001*  .005*  .103 | .357  .188  .587  .439 | .838  .298  .179  .787 |
| Sway Area | Group  Condition Group*Condition | 6.99  40.07  0.97 | 2,120  3,343  6,343 | .001*  <.001*  .447 |  | EO  EC  EOHR  ECHR | 235.30 ± 185.21  369.74 ± 264.32  302.08 ± 217.11  454.61 ± 322.25 | 68  70  72  67 | 177.29 ± 126.11  239.02 ± 144.90  200.22 ± 92.67  324.97 ± 186.30 | 24  24  24  25 | 177.77 ± 101.58  209.64 ± 124.06  138.65 ± 55.50  310.25 ± 173.49 | 26  26  24  25 | .251  .003*  .012*  .009* | .285  .034*  .109  .016* | .999  .783  .760  .990 |
| CI_ml_ | Group  Condition Group*Condition | 7.87  1.01  1.35 | 2,121  3,356  6,356 | <.001*  .388  .234 |  | EO  EC  EOHR  ECHR | 5.14 ± 2.10  5.50 ± 1.81  5.05 ± 1.84  5.19 ± 1.65 | 73  71  72  69 | 5.97 ± 2.70  5.79 ± 1.92  5.59 ± 1.32  5.85 ± 1.14 | 25  25  25  25 | 5.05 ± 2.19  6.62 ± 1.99  6.91 ± 1.92  6.55 ± 1.70 | 26  26  26  26 | .092  .022*  <.001*  .004* | .145  .731  .412  .245 | .987  .258  .036*  .382 |
| CI_ap_ | Group  Condition Group*Condition | 4.45  2.67  2.07 | 2,121  3,356  6,356 | .014*  .048*  .056 |  | EO  EC  EOHR  ECHR | 3.72 ± 1.27  4.04 ± 1.21  3.90 ± 1.41  4.28 ± 1.44 | 73  71  72  69 | 4.16 ± 1.43  3.85 ± 1.00  3.85 ± 0.98  4.18 ± 1.18 | 25  25  25  25 | 4.49 ± 1.31  5.05 ± 1.56  4.63 ± 1.44  4.46 ± 1.49 | 26  26  26  26 | .033*  .003*  .048*  .811 | .334  .818  .981  .954 | .645  .005*  .093  .735 |

*Note*. RMS_ml_ = Root Mean Square medio-lateral; RMS_ap_ = Root Mean Square antero-posterior; RMS_total_ = Root Mean Square for total deviation; Cl_ml_ = Complexity Index medio-lateral; Cl_ap_ = Complexity Index antero-posterior; PS = patients with psychomotor slowing; non-PS = patients without psychomotor slowing; HC = healthy controls; EO = eyes open, natural upright head position, and feet hip-width apart; EC = eyes closed natural upright head position, and feet hip-width apart; EOHR = eyes open, head reclined, and feet hip-width apart; ECHR = eyes closed, head reclined, and feet hip-width apart; N = Number of participants for the specific group and condition; sd = standard deviation; btw = between.

* p < 0.05

# ANOVA and Posthocs for postural parameters within patients with and without controlling for Medication

**Methods**

To evaluate the effect of medication, we calculated between patients analyses with and without controlling for medication (OLZ eq.) (Table S8).

**Results**

Medication had no main effect on any postural parameter in the ANOVA including only patients. Posthocs are unchanged.

Table S5. ANOVA and Posthocs including only patients (PS & non-PS), without and with controlling for Medication (OLZ eq.)

|  | Main ANOVAs only patients | | | |  |  | p-value for posthocs  btw groups |  | Main ANOVAs only patients controlling for OLZ eq. | | | |  |  | p-value for posthocs  btw groups |
| --- | --- | --- | --- | --- | --- | --- | --- | --- | --- | --- | --- | --- | --- | --- | --- |
|  |  | F | numDF, denDF | p |  |  | PS vs. non-PS |  |  | F | numDF, denDF | p |  |  | PS vs. non-PS |
| RMS_ml_ | Group  Condition Group*Condition | 8.33  4.76  0.36 | 1,96  3,277  3,277 | .005*  .003*  .783 | Conditions | EO  EC  EOHR  ECHR | .022*  .033*  .018*  .004* |  | Group  Condition  OLZ eq. Group*Condition | 8.38  4.76  1.45  0.36 | 1,95  3,277  1,95  3,277 | .005*  .003*  .232  .786 | Conditions | EO  EC  EOHR  ECHR | .021*  .031*  .017*  .003* |
| RMS_ap_ | Group  Condition Group*Condition | .85  34.74  0.33 | 1,96  3,277  3,277 | .360  <.001*  .805 |  | EO  EC  EOHR  ECHR | .489  .199  .818  .539 |  | Group  Condition  OLZ eq. Group*Condition | 0.85  34.74  0.87  0.32 | 1,95  3,277  1,95  3,277 | .360  <.001*  .353  .808 |  | EO  EC  EOHR  ECHR | .500  .209  .834  .551 |
| RMS_total_ | Group  Condition Group*Condition | 2.67  30.74  0.19 | 1,96  3,277  3,277 | .105  <.001*  .905 |  | EO  EC  EOHR  ECHR | .205  .094  .346  .197 |  | Group  Condition  OLZ eq. Group*Condition | 2.65  30.75  0.10  0.19 | 1,95  3,277  1,95  3,277 | .107  <.001*  .750  .906 |  | EO  EC  EOHR  ECHR | .208  .097  .351  .200 |
| Sway Area | Group  Condition Group*Condition | 6.38  30.09  0.70 | 1,96  3,274  3,274 | .013*  <.001*  .550 |  | EO  EC  EOHR  ECHR | 0.142  .017*  .054  .009* |  | Group  Condition  OLZ eq. Group*Condition | 6.32  30.10  0.21  0.71 | 1,95  3,274  1,95  3,274 | .014*  <.001*  .646  .550 |  | EO  EC  EOHR  ECHR | .140  .017*  .054  .009* |
| CI_ml_ | Group  Condition Group*Condition | 3.14  1.37  0.52 | 1,96  3,285  3,285 | .080  .251  .667 |  | EO  EC  EOHR  ECHR | .058  .457  .197  .100 |  | Group  Condition  OLZ eq. Group*Condition | 3.12  1.37  0.32  0.52 | 1,95  3,285  1,95  3,285 | .081  .251  .573  .667 |  | EO  EC  EOHR  ECHR | .057  .451  .193  .099 |
| CI_ap_ | Group  Condition Group*Condition | 0.03  3.04  1.60 | 1,96  3,285  3,285 | .869  .029*  .189 |  | EO  EC  EOHR  ECHR | .148  .528  .850  .885 |  | Group  Condition  OLZ eq. Group*Condition | 0.03  3.04  2.04  1.60 | 1,95  3,285  1,95  3,285 | .868  .029*  .157  .189 |  | EO  EC  EOHR  ECHR | .156  .508  .825  .862 |

*Note*. RMS_ml_ = Root Mean Square medio-lateral; RMS_ap_ = Root Mean Square antero-posterior; RMS_total_ = Root Mean Square for total deviation; Cl_ml_ = Complexity Index medio-lateral; Cl_ap_ = Complexity Index antero-posterior; PS = patients with psychomotor slowing; non-PS = patients without psychomotor slowing; HC = healthy controls; EO = eyes open, natural upright head position, and feet hip-width apart; EC = eyes closed natural upright head position, and feet hip-width apart; EOHR = eyes open, head reclined, and feet hip-width apart; ECHR = eyes closed, head reclined, and feet hip-width apart; OLZ eq. = olanzapine equivalent in mg/day; btw = between.

* p < 0.05

# ANOVA and Posthocs for postural parameters including factors “vision” and “vestibular”

**Methods**

Given that our experimental conditions involve crossing visual and vestibular manipulations (i.e., closing the eyes and reclining the head), we aimed to assess the effects of these restrictions between groups across conditions. We defined a "vision" factor, coded as 0 when there was no visual manipulation (EO, EOHR) and 1 when vision was restricted by closing the eyes (EC, ECHR). Similarly, we defined a "vestibular" factor, coded as 0 when the head was upright (EO, EC) and 1 when the head was tilted back (EOHR, ECHR).

We performed two two-way ANOVAs: one including the predictors "group" and "vision" (Table S6), and another with "group" and "vestibular" (Table S7).

Additionally, we conducted a third three-way ANOVA incorporating all three predictors: "group," "vision," and "vestibular" (Table S8). This analysis focused on examining the crossover effects of visual and vestibular manipulations. These analyses allowed us to investigate the effects of visual restriction and vestibular challenge, respectively, across our predefined conditions (EO, EC, EOHR, ECHR).

**Results**

In the two-way ANOVAs, there were no significant interactions between "group" and "vision" or "group" and "vestibular." Regarding the main effects, visual restriction had a considerably greater impact on balance maintenance compared to vestibular challenges. Post hoc analyses revealed that PS participants exhibited significant worsening in balance-related parameters (RMS, sway area) when either closing their eyes or tilting their heads back. Non-PS and HC participants also showed some decline under these manipulations, though not consistently across all parameters. Notably, the complexity index remained largely unaffected by either visual or vestibular manipulations.

In the three-way ANOVA, only one triple interaction was observed, which may not withstand correction for multiple comparisons. There was a significant interaction between "group" and "vestibular" for sway area. Similar to the two-way analyses, the complexity index appeared mostly unaffected, even under combined visual and vestibular manipulations. The visual manipulation had a greater impact than the vestibular challenge, and the combined manipulation yielded the most significant post hoc results across all three groups, likely due to the loss of visual compensation for vestibular challenges or vice-versa.

Table S6. ANOVA and Posthocs for predictors “group” and “vision”

|  | Main ANOVAs | | | |  |  | PS |  | non-PS |  | HC |  |  |  | Posthocs per group (p-value) | | |
| --- | --- | --- | --- | --- | --- | --- | --- | --- | --- | --- | --- | --- | --- | --- | --- | --- | --- |
|  |  | F | DF | p |  |  | mean ± sd | N | mean ± sd | N | mean ± sd | N |  | Difference | PS | non-PS | HC |
| RMS_ml_ | Group  Vision Group*Vision | 9.04  7.38  0.20 | 2,122  1,359  2,359 | <.001*  .007  .822 | Descriptive | eyes open | 2.95 ± 1.71 | 73 | 2.09 ± 1.03 | 25 | 1.98 ± 0.92 | 27 | Post-hocs | eyes open - eyes closed | .014* | .312 | .410 |
|  |  |  |  |  |  | eyes closed | 3.15 ± 1.69 | 73 | 2.25 ± 1.08 | 25 | 2.11 ± 0.96 | 27 |  |  |  |  |  |
| RMS_ap_ | Group  Vision Group*Vision | 4.95  83.12  0.24 | 2,122  1,359  2,359 | .001*  <.001*  .789 |  | eyes open | 5.40 ± 2.17 | 73 | 5.15 ± 1.88 | 25 | 4.34 ± 1.35 | 27 |  | eyes open - eyes closed | <.001* | <.001* | <.001* |
|  |  |  |  |  |  | eyes closed | 6.92 ± 2.53 | 73 | 6.43 ± 2.31 | 25 | 5.72 ± 2.11 | 27 |  |  |  |  |  |
| RMS_total_ | Group  Vision Group*Vision | 6.92  74.47  0.18 | 2,122  1,359  2,359 | .001*  <.001*  .838 |  | eyes open | 6.29 ± 2.44 | 73 | 5.64 ± 1.89 | 25 | 4.85 ± 1.38 | 27 |  | eyes open - eyes closed | <.001* | <.001* | <.001* |
|  |  |  |  |  |  | eyes closed | 7.70 ± 2.78 | 73 | 6.88 ± 2.36 | 25 | 6.17 ± 2.13 | 27 |  |  |  |  |  |
| Sway area | Group  Vision Group*Vision | 7.57  89.37  1.57 | 2,121  1,352  2,352 | <.001*  <.001*  0.208 |  | eyes open | 269.6 ± 204.3 | 73 | 188.8 ± 110.1 | 25 | 159.0 ± 84.3 | 26 |  | eyes open - eyes closed | <.001* | <.001* | .001* |
|  |  |  |  |  |  | eyes closed | 420.6 ± 303.9 | 73 | 282.9 ± 171.2 | 25 | 259.0 ± 157.2 | 26 |  |  |  |  |  |
| CI_ml_ | Group  Vision Group*Vision | 7.93  1.83  0.19 | 2,121  1,366  2,366 | <.001*  .177  .824 |  | eyes open | 5.10 ± 1.97 | 73 | 5.78 ± 2.11 | 25 | 6.48 ± 2.09 | 26 |  | eyes open - eyes closed | .157 | .873 | .671 |
|  |  |  |  |  |  | eyes closed | 5.32 ± 1.72 | 73 | 5.82 ± 1.56 | 25 | 6.59 ± 1.83 | 26 |  |  |  |  |  |
| CI_ap_ | Group  Vision Group*Vision | 4.53  6.64  0.92 | 2,121  1, 366  2,366 | .013*  .010*  .401 |  | eyes open | 3.81 ± 1.34 | 73 | 4.00 ± 1.22 | 25 | 4.56 ± 1.37 | 26 |  | eyes open - eyes closed | .007* | .956 | .317 |
|  |  |  |  |  |  | eyes closed | 4.13 ± 1.33 | 73 | 4.02 ± 1.10 | 25 | 4.76 ± 1.54 | 26 |  |  |  |  |  |

*Note*. RMS_ml_ = Root Mean Square medio-lateral; RMS_ap_ = Root Mean Square antero-posterior; RMS_total_ = Root Mean Square for total deviation; Cl_ml_ = Complexity Index medio-lateral; Cl_ap_ = Complexity Index antero-posterior; PS = patients with psychomotor slowing; non-PS = patients without psychomotor slowing; HC = healthy controls.

* p < 0.05

Table S7. ANOVA and Posthocs for predictors “group” and “vestibular”

|  | Main ANOVAs | | | |  |  | PS |  | non-PS |  | HC |  |  |  | Posthocs per group (p-value) | | |
| --- | --- | --- | --- | --- | --- | --- | --- | --- | --- | --- | --- | --- | --- | --- | --- | --- | --- |
|  |  | F | DF | p |  |  | mean ± sd | N | mean ± sd | N | mean ± sd | N |  | Difference | PS | non-PS | HC |
| RMS_ml_ | Group  Vestibular  Group* Vestibular | 9.07  2.98  2.95 | 2,122  1,359  2,359 | <.001*  .085  .053 | Descriptive | head upright | 2.92 ± 1.72 | 73 | 2.10 ± 1.19 | 25 | 2.17 ± 1.04 | 27 | Post-hocs | head upright - reclined | .009* | .511 | .219 |
|  |  |  |  |  |  | head reclined | 3.17 ± 1.69 | 72 | 2.24 ± 0.91 | 25 | 1.92 ± 0.80 | 27 |  |  |  |  |  |
| RMS_ap_ | Group  Vestibular  Group* Vestibular | 5.00  39.47  0.37 | 2,122  1,359  2,359 | .008*  <.001*  .690 |  | head upright | 5.68 ± 2.13 | 73 | 5.13 ± 1.81 | 25 | 4.46 ± 1.42 | 27 |  | head upright - reclined | <.001* | .001* | <.001* |
|  |  |  |  |  |  | head reclined | 6.63 ± 2.68 | 72 | 6.42 ± 2.35 | 25 | 5.62 ± 2.14 | 27 |  |  |  |  |  |
| RMS_total_ | Group  Vestibular  Group* Vestibular | 6.99  34.86  0.14 | 2,122  1,359  2,359 | .001  <.001*  .872 |  | head upright | 6.52 ± 2.40 | 73 | 5.65 ± 1.88 | 25 | 5.03 ± 1.53 | 27 |  | head upright - reclined | <.001* | .002* | .005* |
|  |  |  |  |  |  | head reclined | 7.45 ± 2.91 | 72 | 6.86 ± 2.36 | 25 | 5.99 ± 2.14 | 27 |  |  |  |  |  |
| Sway area | Group  Vestibular  Group* Vestibular | 7.44  15.03  0.17 | 2,121  1,352  2,352 | <.001*  <.001*  .846 |  | head upright | 312.1 ± 252.0 | 73 | 208.2 ± 137.9 | 25 | 193.7 ± 113.4 | 26 |  | head upright - reclined | <.001* | .130 | .168 |
|  |  |  |  |  |  | head reclined | 378.3 ± 282.8 | 72 | 263.9 ± 159.5 | 25 | 226.2 ± 155.0 | 25 |  |  |  |  |  |
| CI_ml_ | Group  Vestibular  Group* Vestibular | 7.91  0.44  2.19 | 2,121  1,366  2,366 | <.001*  .507  .113 |  | head upright | 5.30 ± 1.95 | 73 | 5.88 ± 2.32 | 25 | 6.34 ± 2.09 | 26 |  | head upright - reclined | .150 | .547 | .123 |
|  |  |  |  |  |  | head reclined | 5.11 ± 1.74 | 72 | 5.72 ± 1.23 | 25 | 6.73 ± 1.80 | 26 |  |  |  |  |  |
| CI_ap_ | Group  Vestibular  Group* Vestibular | 4.52  0.46  1.67 | 2,121  1, 366  2,366 | .013  .497  .190 |  | head upright | 3.88 ± 1.24 | 73 | 4.00 ± 1.23 | 25 | 4.77 ± 1.45 | 26 |  | head upright - reclined | .120 | .960 | .244 |
|  |  |  |  |  |  | head reclined | 4.06 ± 1.44 | 72 | 4.01 ± 1.09 | 25 | 4.55 ± 1.45 | 26 |  |  |  |  |  |

*Note*. RMS_ml_ = Root Mean Square medio-lateral; RMS_ap_ = Root Mean Square antero-posterior; RMS_total_ = Root Mean Square for total deviation; Cl_ml_ = Complexity Index medio-lateral; Cl_ap_ = Complexity Index antero-posterior; PS = patients with psychomotor slowing; non-PS = patients without psychomotor slowing; HC = healthy controls.

* p < 0.05

Table S8. ANOVA and Posthocs for predictors “group”, “vision” and “vestibular”

|  | Main ANOVAs | | | |  |  |  | PS |  | non-PS |  | HC |  |  |  |  | Posthoc differences per group (p-value) | | |
| --- | --- | --- | --- | --- | --- | --- | --- | --- | --- | --- | --- | --- | --- | --- | --- | --- | --- | --- | --- |
|  |  | F | DF | p |  |  |  | mean ± sd | N | mean ± sd | N | mean ± sd | N |  |  | Difference between: | PS | non-PS | HC |
| RMS_medio-lateral_ | Group  Vision  Vestibular  Vision*Vestibular  Group*Vision  Group*Vestibular Group*Vision*Vestibular | 9.03  7.54  3.05  0.20  3.02  3.51  0.29 | 2,122  1,353  1,353  2,353  2,353  1,353  2,353 | <.001*  .006*  .082  .817  .050  .062  .751 | Descriptive | head upright | eyes open | 2.92 ± 1.88 | 70 | 2.04 ± 1.27 | 24 | 2.20 ± 1.02 | 27 | Posthocs |  | eyes open - closed | .557 | .559 | .817 |
|  |  |  |  |  |  |  | eyes closed | 2.92 ± 1.55 | 70 | 2.16 ± 1.12 | 24 | 2.15 ± 1.08 | 27 |  | HR | eyes open - closed | .004* | .391 | .154 |
|  |  |  |  |  |  | head reclined | eyes open | 2.98 ± 1.55 | 72 | 2.13 ± 0.75 | 25 | 1.75 ± 0.76 | 26 |  | EO | head upright - reclined | .459 | .734 | .087 |
|  |  |  |  |  |  |  | eyes closed | 3.38 ± 1.80 | 71 | 2.35 ± 1.05 | 25 | 2.08 ± 0.83 | 26 |  | EC | head upright - reclined | .003* | .552 | .969 |
| RMS_antero-posterior_ | Group  Vision  Vestibular  Vision*Vestibular  Group*Vision  Group*Vestibular Group*Vision*Vestibular | 4.95  94.72  50.09  0.28  0.46  0.67  2.12 | 2,122  1,353  1,353  2,353  2,353  1,353  2,353 | .009*  <.001*  <.001*  .754  .631  .414  .122 |  | head upright | eyes open | 4.91 ± 1.85 | 70 | 4.49 ±1.69 | 24 | 4.12 ± 1.23 | 27 |  |  | eyes open - closed | <.001* | .007* | .136 |
|  |  |  |  |  |  |  | eyes closed | 6.45 ± 2.14 | 70 | 5.77 ± 1.73 | 24 | 4.79 ± 1.55 | 27 |  | HR | eyes open - closed | <.001* | .006* | <.001* |
|  |  |  |  |  |  | head reclined | eyes open | 5.88 ± 2.35 | 72 | 5.77 ± 1.87 | 25 | 4.56 ± 1.46 | 26 |  | EO | head upright - reclined | <.001* | .009* | .258 |
|  |  |  |  |  |  |  | eyes closed | 7.39 ± 2.80 | 71 | 7.07 ± 2.63 | 25 | 6.69 ± 2.20 | 26 |  | EC | head upright - reclined | .002* | .009* | <.001* |
| RMS_total_ | Group  Vision  Vestibular  Vision*Vestibular  Group*Vision  Group*Vestibular Group*Vision*Vestibular | 6.91  83.6  43.37  0.21  0.16  2.05  1.68 | 2,122  1,353  1,353  2,353  2,353  1,353  2,353 | .001*  <.001*  <.001*  .812  .855  .154  .189 |  | head upright | eyes open | 5.87 ± 2.27 | 70 | 5.08 ± 1.74 | 24 | 4.75 ± 1.35 | 27 |  |  | eyes open - closed | <.001* | .017* | .211 |
|  |  |  |  |  |  |  | eyes closed | 7.17 ± 2.37 | 70 | 6.21 ± 1.88 | 24 | 5.32 ± 1.68 | 27 |  | HR | eyes open - closed | <.001* | .005* | <.001* |
|  |  |  |  |  |  | head reclined | eyes open | 6.70 ± 2.55 | 72 | 6.19 ± 1.90 | 25 | 4.95 ± 1.44 | 26 |  | EO | head upright - reclined | .002* | .026* | .534 |
|  |  |  |  |  |  |  | eyes closed | 8.22 ± 3.06 | 71 | 7.53 ± 2.62 | 25 | 7.04 ± 2.23 | 26 |  | EC | head upright - reclined | <.001* | .010* | <.001* |
| Sway area | Group  Vision  Vestibular  Vision*Vestibular  Group*Vision  Group*Vestibular Group*Vision*Vestibular | 7.50  95.50  20.35  1.83  0.35  6.11  0.91 | 2,121  1,346  1,346  2,346  2,346  1,346  2,346 | .001*  <.001*  <.001*  .162  .706  .014*  .406 |  | head upright | eyes open | 235.3 ± 185 | 68 | 177.3 ± 126 | 24 | 177.8 ± 101 | 26 |  |  | eyes open - closed | <.001* | .081 | .409 |
|  |  |  |  |  |  |  | eyes closed | 384.7 ± 284 | 72 | 239.0 ± 144 | 24 | 209.6 ± 124 | 26 |  | HR | eyes open - closed | <.001* | .002* | <.001* |
|  |  |  |  |  |  | head reclined | eyes open | 302.1 ± 217 | 72 | 200.2 ± 92 | 24 | 138.6 ± 55 | 24 |  | EO | head upright - reclined | .053 | .632 | .585 |
|  |  |  |  |  |  |  | eyes closed | 457.9 ± 320 | 69 | 325.0 ± 186 | 25 | 310.3 ± 173 | 25 |  | EC | head upright - reclined | <.001* | .063 | .007* |
| CI_medio-lateral_ | Group  Vision  Vestibular  Vision*Vestibular  Group*Vision  Group*Vestibular Group*Vision*Vestibular | 7.91  1.85  0.44  0.19  2.19  1.01  1.76 | 2,121  1,360  1,360  2,360  2,360  1,360  2,360 | .001*  .175  .509  .824  .113  .316  .174 |  | head upright | eyes open | 5.14 ± 2.10 | 73 | 5.97 ± 2.70 | 25 | 6.05 ± 2.19 | 26 |  |  | eyes open - closed | .131 | .629 | .113 |
|  |  |  |  |  |  |  | eyes closed | 5.47 ± 1.80 | 73 | 5.79 ± 1.92 | 25 | 6.62 ± 1.99 | 26 |  | HR | eyes open - closed | .627 | .478 | .327 |
|  |  |  |  |  |  | head reclined | eyes open | 5.05 ±1.84 | 72 | 5.59 ± 1.32 | 25 | 6.91 ± 1.92 | 26 |  | EO | head upright - reclined | .608 | .307 | .018* |
|  |  |  |  |  |  |  | eyes closed | 5.18 ± 1.63 | 71 | 5.85 ± 1.14 | 25 | 6.55 ± 1.70 | 26 |  | EC | head upright - reclined | .129 | .865 | .848 |
| CI_antero-posterior_ | Group  Vision  Vestibular  Vision*Vestibular  Group*Vision  Group*Vestibular Group*Vision*Vestibular | 4.52  6.72  0.48  0.93  1.72  0.02  3.15 | 2,121  1,360  1,360  2,360  2,360  1,360  2,360 | .013*  .010*  .488  .396  .180  .883  .044* |  | head upright | eyes open | 3.72 ± 1.27 | 73 | 4.16 ± 1.43 | 25 | 4.49 ± 1.31 | 26 |  |  | eyes open - closed | .051 | .266 | .040* |
|  |  |  |  |  |  |  | eyes closed | 4.04 ± 1.19 | 73 | 3.85 ±1.00 | 25 | 5.05 ± 1.56 | 26 |  | HR | eyes open - closed | .054 | .234 | .522 |
|  |  |  |  |  |  | head reclined | eyes open | 3.90 ± 1.41 | 72 | 3.85 ± 0.98 | 25 | 4.63 ± 1.44 | 26 |  | EO | head upright - reclined | .259 | .264 | .604 |
|  |  |  |  |  |  |  | eyes closed | 4.22 ± 1.46 | 71 | 4.18 ± 1.18 | 25 | 4.46 ± 1.49 | 26 |  | EC | head upright - reclined | .263 | .235 | .029* |

*Note*. RMS_ml_ = Root Mean Square medio-lateral; RMS_ap_ = Root Mean Square antero-posterior; RMS_total_ = Root Mean Square for total deviation; Cl_ml_ = Complexity Index medio-lateral; Cl_ap_ = Complexity Index antero-posterior; PS = patients with psychomotor slowing; non-PS = patients without psychomotor slowing; HC = healthy controls

* p < 0.05

# Point Plot for associations of postural parameters with motor scales, activity level, and BNSS for condition EO

Figure S3. Correlation between postural parameters and clinical rating scales across all patients

*Note*. Large dots indicate high absolute correlation coefficients. Color indicates direction of correlation (blue = negative correlation, red = positive correlation). Cross indicates not significant correlations.

RMS_ml_ = Root Mean Square medio-lateral; RMS_ap_ = Root Mean Square antero-posterior; RMS_total_ = Root Mean Square for total deviation; Cl_ml_ = Complexity Index medio-lateral; Cl_ap_ = Complexity Index antero-posterior; mSRRS = motor part of the SRRS; UPDRS = Unified Parkinson Disease Rating Scale Part III; BFCRS = Bush-Francis Catatonia Rating Scale; NES = Neurological Evaluation Scale; BNSS = Brief Negative Symptom Scale.

# Associations of postural parameters with motor scales, activity level, and BNSS for EO conditions separated into PS and non-PS

Table S9. Associations of postural parameters with motor scales, activity level, and BNSS for condition EO separately in PS and non-PS

|  |  | RMS_ml_ | | | RMS_ap_ | | | RMS_total_ | | | Sway Area | | | CI_ml_ | | | CI_ap_ | | |
| --- | --- | --- | --- | --- | --- | --- | --- | --- | --- | --- | --- | --- | --- | --- | --- | --- | --- | --- | --- |
|  | Group | N | Tau | p | N | Tau | p | N | Tau | p | N | Tau | p | N | Tau | p | N | Tau | p |
| mSRRS | PS | 70 | .110 | .195 | 70 | .132 | .121 | 70 | .166 | .051 | 68 | .078 | .365 | 73 | -.181 | .029* | 73 | -.238 | .004* |
|  | non-PS | 24 | .092 | .563 | 24 | .100 | .528 | 24 | .083 | .599 | 24 | .150 | .343 | 25 | -.111 | .475 | 25 | -.034 | .824 |
| UPDRS | PS | 70 | .160 | .053 | 70 | .211 | .011* | 70 | .215 | .009* | 68 | .127 | .132 | 73 | -.175 | .030* | 73 | -.166 | .040* |
|  | non-PS | 24 | .111 | .455 | 24 | .052 | .727 | 24 | .133 | .370 | 24 | .200 | .179 | 25 | .031 | .833 | 25 | .031 | .833 |
| BFCRS | PS | 70 | .122 | .150 | 70 | .070 | .409 | 70 | .119 | .159 | 68 | .068 | .433 | 73 | -.154 | .064 | 73 | -.011 | .897 |
|  | non-PS | 24 | .323 | .045* | 24 | -.043 | .792 | 24 | .094 | .562 | 24 | .238 | .140 | 25 | -.253 | .110 | 25 | .024 | .881 |
| NES total | PS | 67 | .130 | .126 | 67 | .206 | .015* | 67 | .224 | .008* | 65 | .128 | .137 | 70 | -.133 | .108 | 70 | -.090 | .275 |
|  | non-PS | 24 | .176 | .240 | 24 | .214 | .154 | 24 | .274 | .068 | 24 | .274 | .068 | 25 | -.166 | .259 | 25 | -.083 | .572 |
| NES sensory integration | PS | 67 | .182 | .041* | 67 | .162 | .068 | 67 | .194 | .029* | 65 | .198 | .028* | 70 | -.146 | .093 | 70 | .000 | .996 |
|  | non-PS | 24 | .141 | .383 | 24 | -.021 | .895 | 24 | .055 | .731 | 24 | .252 | .119 | 25 | .067 | .671 | 25 | .123 | .438 |
| NES motor coordination | PS | 68 | .128 | .153 | 68 | .121 | .176 | 68 | .174 | .053 | 66 | .092 | .312 | 71 | -.117 | .181 | 71 | -.079 | .368 |
|  | non-PS | 24 | .432 | .010* | 24 | .356 | .035* | 24 | .365 | .030* | 24 | .517 | .002* | 25 | -.429 | .008* | 25 | -.166 | .309 |
| NES sequencing | PS | 67 | .093 | .284 | 67 | .189 | .029* | 67 | .210 | .015* | 65 | .074 | .402 | 70 | -.079 | .354 | 70 | -.044 | .603 |
|  | non-PS | 24 | .004 | .980 | 24 | .153 | .323 | 24 | .192 | .214 | 24 | .114 | .462 | 25 | -.068 | .651 | 25 | -.018 | .905 |
| NES others | PS | 67 | .129 | .137 | 67 | .166 | .056 | 67 | .178 | .040* | 65 | .151 | .088 | 70 | -.143 | .091 | 70 | -.129 | .127 |
|  | non-PS | 24 | .112 | .454 | 24 | .157 | .294 | 24 | .210 | .162 | 24 | .150 | .318 | 25 | -.048 | .742 | 25 | -.132 | .371 |
| Activity level | PS | 63 | -.075 | .383 | 63 | -.132 | .127 | 63 | -.111 | .198 | 62 | -.091 | .293 | 66 | .115 | .172 | 66 | .240 | .004* |
|  | non-PS | 23 | -.012 | .958 | 23 | -.154 | .319 | 23 | -.067 | .676 | 23 | -.043 | .794 | 24 | -.022 | .902 | 24 | .058 | .713 |
| BNSS total | PS | 70 | .156 | .058 | 70 | .067 | .414 | 70 | .104 | .206 | 68 | .137 | .103 | 73 | -.241 | .003* | 73 | -.108 | .182 |
|  | non-PS | 24 | .285 | .053 | 24 | .153 | .297 | 24 | .161 | .275 | 24 | .285 | .053 | 25 | -.124 | .387 | 25 | -.198 | .168 |
| BNSS Anhedonia | PS | 70 | .131 | .120 | 70 | .026 | .756 | 70 | .052 | .538 | 68 | .072 | .398 | 73 | -.203 | .014* | 73 | -.026 | .749 |
|  | non-PS | 24 | .196 | .194 | 24 | .203 | .177 | 24 | .181 | .230 | 24 | .278 | .064 | 25 | -.086 | .556 | 25 | -.197 | .180 |
| BNSS Distress | PS | 70 | .146 | .098 | 70 | .042 | .637 | 70 | .055 | .531 | 68 | .041 | .649 | 73 | -.266 | .002* | 73 | -.090 | .298 |
|  | non-PS | 24 | .202 | .194 | 24 | .099 | .525 | 24 | .154 | .321 | 24 | .265 | .088 | 25 | -.040 | .792 | 25 | -.157 | .303 |
| BNSS Asocial | PS | 70 | .151 | .080 | 70 | .081 | .346 | 70 | .113 | .189 | 68 | .136 | .120 | 73 | -.135 | .110 | 73 | .025 | .765 |
|  | non-PS | 24 | .147 | .338 | 24 | .085 | .579 | 24 | .070 | .650 | 24 | .147 | .338 | 25 | -.163 | .275 | 25 | -.206 | .169 |
| BNSS Avolition | PS | 70 | .182 | .035* | 70 | .039 | .655 | 70 | .085 | .326 | 68 | .147 | .094 | 73 | -.203 | .016* | 73 | -.008 | .923 |
|  | non-PS | 24 | .156 | .303 | 24 | .126 | .407 | 24 | .141 | .353 | 24 | .187 | .219 | 25 | -.155 | .298 | 25 | -.190 | .202 |
| BNSS Affect | PS | 70 | .161 | .056 | 70 | .122 | .148 | 70 | .142 | .091 | 68 | .174 | .042* | 73 | -.210 | .011* | 73 | -.186 | .024* |
|  | non-PS | 24 | .182 | .229 | 24 | .046 | .764 | 24 | .076 | .616 | 24 | .250 | .098 | 25 | .097 | .509 | 25 | -.083 | .572 |
| BNSS Alogia | PS | 70 | .077 | .366 | 70 | .009 | .915 | 70 | .017 | .842 | 68 | .135 | .119 | 73 | -.191 | .022* | 73 | -.092 | .272 |
|  | non-PS | 24 | .124 | .437 | 24 | .013 | .936 | 24 | .081 | .611 | 24 | .090 | .574 | 25 | .012 | .939 | 25 | .004 | .980 |

*Note*. mSRRS = motor part of the SRRS; SRRS = Salpêtrière Retardation Rating Scale; UPDRS = Unified Parkinson Disease Rating Scale Part III; BFCRS = Bush-Francis Catatonia Rating Scale; NES = Neurological Evaluation Scale; BNSS = Brief Negative Symptom Scale; PS = patients with psychomotor slowing; non-PS = patients without psychomotor slowing; HC = healthy controls; RMS_ml_ = Root Mean Square medio-lateral; RMS_ap_ = Root Mean Square antero-posterior; RMS_total_ = Root Mean Square for total deviation; Cl_ml_ = Complexity Index medio-lateral; Cl_ap_ = Complexity Index antero-posterior; N = Number of participants for the specific postural parameter and the clinical or motor scale. * p < 0.05

# Associations of postural parameters with motor scales, activity level, and BNSS for conditions EC, EOHR, and ECHR

Table S10. Associations of postural parameters with motor scales, activity level, and BNSS for condition EC

|  | RMS_ml_ | | | RMS_ap_ | | | RMS_total_ | | | Sway Area | | | CI_ml_ | | | CI_ap_ | | |
| --- | --- | --- | --- | --- | --- | --- | --- | --- | --- | --- | --- | --- | --- | --- | --- | --- | --- | --- |
|  | N | Tau | p | N | Tau | p | N | Tau | p | N | Tau | p | N | Tau | p | N | Tau | p |
| mSRRS | 94 | .210 | .003* | 94 | .093 | .198 | 94 | .144 | .046* | 96 | .179 | .692 | 98 | -.178 | .012 | 98 | -.023 | .739 |
| SRRS | 94 | .210 | .003* | 94 | .152 | .033* | 94 | .187 | .009* | 96 | .182 | .010* | 98 | -.109 | .118 | 98 | -.006 | .934 |
| UPDRS | 94 | .201 | .005* | 94 | .124 | .081 | 94 | .177 | .012* | 96 | .204 | .004* | 98 | -.184 | .008* | 98 | -.012 | .866 |
| BFCRS | 94 | .185 | .012* | 94 | .078 | .289 | 94 | .124 | .092* | 96 | .139 | .054 | 98 | -.095 | .183 | 98 | -.051 | .477 |
| NES total | 91 | .096 | .185 | 91 | .120 | .098 | 91 | .132 | .069 | 93 | .149 | .038* | 95 | -.098 | .165 | 95 | -.046 | .517 |
| NES sensory integration | 91 | .113 | .138 | 91 | .034 | .658 | 91 | .052 | .023* | 93 | .110 | .146 | 95 | -.078 | .297 | 95 | .105 | .157 |
| NES motor coordination | 92 | .129 | .096 | 92 | .159 | .040* | 92 | .176 | .023* | 94 | .198 | .010* | 96 | -.115 | .128 | 96 | -.060 | .425 |
| NES sequencing | 91 | .054 | .468 | 91 | .089 | .232 | 91 | .097 | .192 | 93 | .118 | .107 | 95 | -.048 | .505 | 95 | .005 | .948 |
| NES others | 91 | .074 | .313 | 91 | .113 | .127 | 91 | .124 | .092 | 93 | .131 | .073 | 95 | -.079 | .273 | 95 | -.122 | .091 |
| Activity level | 87 | -.222 | .002* | 87 | -.111 | .126 | 87 | -.172 | .018* | 88 | -.159 | .028* | 90 | .121 | .092 | 90 | .059 | .413 |
| BNSS total | 94 | .228 | .001* | 94 | .166 | .019* | 94 | .207 | .003* | 96 | .198 | .005* | 98 | -.124 | .072 | 98 | -.032 | .647 |
| BNSS Anhedonia | 94 | .198 | .005* | 94 | .205 | .004* | 94 | .235 | .001 | 96 | .213 | .003* | 98 | -.115 | .103 | 98 | -.032 | .649 |
| BNSS Distress | 94 | .169 | .025* | 94 | .124 | .099 | 94 | .149 | .048* | 96 | .122 | .103 | 98 | -.106 | .152 | 98 | .011 | .881 |
| BNSS Asocial | 94 | .170 | .021* | 94 | .097 | .189 | 94 | .130 | .078 | 96 | .108 | .139 | 98 | -.104 | .149 | 98 | .043 | .556 |
| BNSS Avolition | 94 | .166 | .024* | 94 | .089 | .227 | 94 | .132 | .073 | 96 | .152 | .038* | 98 | -.124 | .087 | 98 | -.008 | .916 |
| BNSS Affect | 94 | .257 | <.001* | 94 | .139 | .054 | 94 | .190 | .008* | 96 | .210 | .003* | 98 | -.168 | .017* | 98 | -.069 | .326 |
| BNSS Alogia | 94 | .099 | .179 | 94 | .063 | .389 | 94 | .079 | .284 | 96 | .082 | .262 | 98 | -.004 | .958 | 98 | .006 | .938 |

*Note*. mSRRS = motor part of the SRRS; SRRS = Salpêtrière Retardation Rating Scale; UPDRS = Unified Parkinson Disease Rating Scale Part III; BFCRS = Bush-Francis Catatonia Rating Scale; NES = Neurological Evaluation Scale; BNSS = Brief Negative Symptom Scale; RMS_ml_ = Root Mean Square medio-lateral; RMS_ap_ = Root Mean Square antero-posterior; RMS_total_ = Root Mean Square for total deviation; Cl_ml_ = Complexity Index medio-lateral; Cl_ap_ = Complexity Index antero-posterior; N = Number of participants for the specific postural parameter and the clinical or motor scale.

* p < 0.05

Table S11. Associations of postural parameters with motor scales, activity level, and BNSS for condition EOHR

|  | RMS_ml_ | | | RMS_ap_ | | | RMS_total_ | | | Sway Area | | | CI_ml_ | | | CI_ap_ | | |
| --- | --- | --- | --- | --- | --- | --- | --- | --- | --- | --- | --- | --- | --- | --- | --- | --- | --- | --- |
|  | N | Tau | p | N | Tau | p | N | Tau | p | N | Tau | p | N | Tau | p | N | Tau | p |
| mSRRS | 97 | .213 | .003* | 97 | .037 | .601 | 97 | .106 | .134 | 96 | .167 | .019* | 97 | -.208 | .003* | 97 | -.065 | .359 |
| SRRS | 97 | .218 | .002* | 97 | .091 | .194 | 97 | .152 | .030* | 96 | .175 | .013* | 97 | -.185 | .008* | 97 | -.134 | .056 |
| UPDRS | 97 | .206 | .003* | 97 | .076 | .278 | 97 | .135 | .054 | 96 | .162 | .021* | 97 | -.207 | .003* | 97 | .011 | .876 |
| BFCRS | 97 | .188 | .009* | 97 | -.007 | .917 | 97 | .053 | .460 | 96 | .099 | .173 | 97 | -.180 | .013* | 97 | .011 | .878 |
| NES total | 94 | .088 | .219 | 94 | .138 | .053 | 94 | .146 | .041* | 93 | .119 | .096 | 94 | -.068 | .343 | 94 | -.049 | .492 |
| NES sensory integration | 94 | .143 | .057 | 94 | .161 | .031* | 94 | .161 | .032* | 93 | .167 | .026* | 94 | -.102 | .172 | 94 | -.051 | .500 |
| NES motor coordination | 95 | .122 | .109 | 95 | .153 | .044* | 95 | .171 | .024* | 94 | .159 | .038* | 95 | -.096 | .208 | 95 | -.071 | .348 |
| NES sequencing | 94 | .001 | .990 | 94 | .103 | .159 | 94 | .091 | .212 | 93 | .065 | .376 | 94 | .057 | .434 | 94 | .003 | .963 |
| NES others | 94 | .095 | .190 | 94 | .073 | .313 | 94 | .094 | .197 | 93 | .073 | .315 | 94 | -.080 | .271 | 94 | -.043 | .557 |
| Activity level | 89 | -.221 | .002* | 89 | -.088 | .223 | 89 | -.157 | .029* | 89 | -.173 | .016* | 89 | .175 | .015* | 89 | .117 | .103 |
| BNSS total | 97 | .216 | .002* | 97 | .072 | .301 | 97 | .132 | .058 | 96 | -.072 | .298 | 97 | -.188 | .007* | 97 | -.072 | .298 |
| BNSS Anhedonia | 97 | .164 | .021* | 97 | .105 | .138 | 97 | .135 | .056 | 96 | -.093 | .187 | 97 | -.153 | .030* | 97 | -.093 | .187 |
| BNSS Distress | 97 | .140 | .059 | 97 | .053 | .477 | 97 | .097 | .191 | 96 | .000 | 1.00 | 97 | -.151 | .041* | 97 | .000 | 1.00 |
| BNSS Asocial | 97 | .123 | .091 | 97 | .116 | .111 | 97 | .138 | .058 | 96 | -.084 | .249 | 97 | -.097 | .180 | 97 | -.084 | .249 |
| BNSS Avolition | 97 | .217 | .003* | 97 | .058 | .423 | 97 | .109 | .132 | 96 | -.113 | .118 | 97 | -.222 | .002* | 97 | -.113 | .118 |
| BNSS Affect | 97 | .233 | .001* | 97 | .046 | .518 | 97 | .117 | .098 | 96 | -.056 | .429 | 97 | -.165 | .020* | 97 | -.056 | .429 |
| BNSS Alogia | 97 | .115 | .113 | 97 | -.021 | .774 | 97 | .015 | .838 | 96 | -.042 | .560 | 97 | -.065 | .373 | 97 | -.042 | .560 |

*Note*. mSRRS = motor part of the SRRS; SRRS = Salpêtrière Retardation Rating Scale; UPDRS = Unified Parkinson Disease Rating Scale Part III; BFCRS = Bush-Francis Catatonia Rating Scale; NES = Neurological Evaluation Scale; BNSS = Brief Negative Symptom Scale; RMS_ml_ = Root Mean Square medio-lateral; RMS_ap_ = Root Mean Square antero-posterior; RMS_total_ = Root Mean Square for total deviation; Cl_ml_ = Complexity Index medio-lateral; Cl_ap_ = Complexity Index antero-posterior; N = Number of participants for the specific postural parameter and the clinical or motor scale.

* p < 0.05

Table S12. Associations of postural parameters with motor scales, activity level, and BNSS for condition ECHR

|  | RMS_ml_ | | | RMS_ap_ | | | RMS_total_ | | | SwayArea | | | CI_ml_ | | | CI_ap_ | | |
| --- | --- | --- | --- | --- | --- | --- | --- | --- | --- | --- | --- | --- | --- | --- | --- | --- | --- | --- |
|  | N | Tau | p | N | Tau | p | N | Tau | p | N | Tau | p | N | Tau | p | N | Tau | p |
| mSRRS | 96 | .227 | .002* | 96 | .014 | .846 | 96 | .061 | .393 | 94 | .115 | .112 | 96 | -.257 | <.001* | 96 | -.012 | .871 |
| SRRS | 96 | .225 | .001* | 96 | .076 | .281 | 96 | .109 | .122 | 94 | .126 | .077 | 96 | -.211 | .003* | 96 | -.078 | .270 |
| UPDRS | 96 | .285 | <.001* | 96 | .119 | .091 | 96 | .163 | .020* | 94 | .205 | .004* | 96 | -.267 | <.001* | 96 | .012 | .869 |
| BFCRS | 96 | .174 | .016* | 96 | -.003 | .962 | 96 | .038 | .603 | 94 | .067 | .365 | 96 | -.150 | .038* | 96 | .048 | .505 |
| NES total | 93 | .121 | .091 | 93 | .141 | .049* | 93 | .142 | .047* | 91 | .129 | .076 | 93 | -.204 | .004* | 93 | -.033 | .649 |
| NES sensory integration | 93 | .106 | .161 | 93 | .000 | .997 | 93 | .005 | .949 | 91 | .120 | .117 | 93 | -.098 | .196 | 93 | .119 | .113 |
| NES motor coordination | 94 | .133 | .082 | 94 | .177 | .021* | 94 | .181 | .018* | 92 | .155 | .046* | 94 | -.176 | .022* | 94 | -.082 | .282 |
| NES sequencing | 93 | .028 | .703 | 93 | .109 | .136 | 93 | .097 | .188 | 91 | .053 | .475 | 93 | -.055 | .450 | 93 | .040 | .584 |
| NES others | 93 | .134 | .066 | 93 | .138 | .058 | 93 | .139 | .057 | 91 | .111 | .132 | 93 | -.224 | .002* | 93 | -.092 | .207 |
| Activity level | 88 | -.212 | .003* | 88 | -.076 | .293 | 88 | -.130 | .074 | 87 | -.163 | .026* | 96 | .178 | .014* | 88 | .088 | .223 |
| BNSS total | 96 | .224 | .001* | 96 | .095 | .175 | 96 | .135 | .053 | 94 | .145 | .040* | 96 | -.133 | .057 | 96 | -.062 | .372 |
| BNSS Anhedonia | 96 | .184 | .010* | 96 | .167 | .019* | 96 | .184 | .010* | 94 | .158 | .029* | 96 | -.142 | .046* | 96 | -.075 | .295 |
| BNSS Distress | 96 | .163 | .029* | 96 | .088 | .238 | 96 | .116 | .118 | 94 | .120 | .112 | 96 | -.092 | .218 | 96 | .039 | .601 |
| BNSS Asocial | 96 | .097 | .186 | 96 | .064 | .383 | 96 | .083 | .253 | 94 | .106 | .153 | 96 | -.043 | .558 | 96 | .025 | .727 |
| BNSS Avolition | 96 | .172 | .018* | 96 | .130 | .076 | 96 | .149 | .041* | 94 | .129 | .081 | 96 | -.148 | .042* | 96 | -.112 | .126 |
| BNSS Affect | 96 | .242 | .001* | 96 | .068 | .338 | 96 | .122 | .087 | 94 | .175 | .015* | 96 | -.123 | .084 | 96 | .000 | .995 |
| BNSS Alogia | 96 | .123 | .091 | 96 | -.031 | .675 | 96 | .009 | .901 | 94 | .035 | .639 | 96 | -.031 | .671 | 96 | -.017 | .810 |

*Note*. mSRRS = motor part of the SRRS; SRRS = Salpêtrière Retardation Rating Scale; UPDRS = Unified Parkinson Disease Rating Scale Part III; BFCRS = Bush-Francis Catatonia Rating Scale; NES = Neurological Evaluation Scale; BNSS = Brief Negative Symptom Scale; RMS_ml_ = Root Mean Square medio-lateral; RMS_ap_ = Root Mean Square antero-posterior; RMS_total_ = Root Mean Square for total deviation; Cl_ml_ = Complexity Index medio-lateral; Cl_ap_ = Complexity Index antero-posterior; N = Number of participants for the specific postural parameter and the clinical or motor scale.

* p < 0.05

# Associations between activity level and postural parameters across all participants

**Methods**

We ran the correlation between activity level and postural parameters across all participants (PS, non-PS, HC) (Table S13, Figure S4).

**Results**

Including HC in the association between activity level and postural parameter increases the absolute Tau value in RMS_ap_, RMS_total_, and CI_ap_ in comparison to only including patients. Cl_ap_ was already significant when only including patients and is now highly significant, while RMS_ap_ and RMS_total_ newly turn significant. The correlations with RMS_ml,_ sway area, and CI_ml_ are reduced in comparison to only including patients and remain nonsignificant.

Table S13. Correlation between postural parameters and activity level all participants

|  | Activity level | | |
| --- | --- | --- | --- |
|  | N | Tau | p-value |
| RMS_ml_ | 113 | -.091 | .154 |
| RMS_ap_ | 113 | -.156 | .014* |
| RMS_total_ | 113 | -.134 | .035* |
| Sway Area | 111 | -.093 | .150 |
| CI_ml_ | 116 | .118 | .059 |
| CI_ap_ | 116 | .228 | <.001* |

*Note*. RMS_ml_ = Root Mean Square medio-lateral; RMS_ap_ = Root Mean Square antero-posterior;

RMS_total_ = Root Mean Square for total deviation; Cl_ml_ = Complexity Index medio-lateral; Cl_ap_ =

Complexity Index antero-posterior; N = Number of participants for the specific postural parameter.

* p < 0.05

Figure S4: Correlations between postural parameters and activity level across patients (top) and across all participants (bottom)*
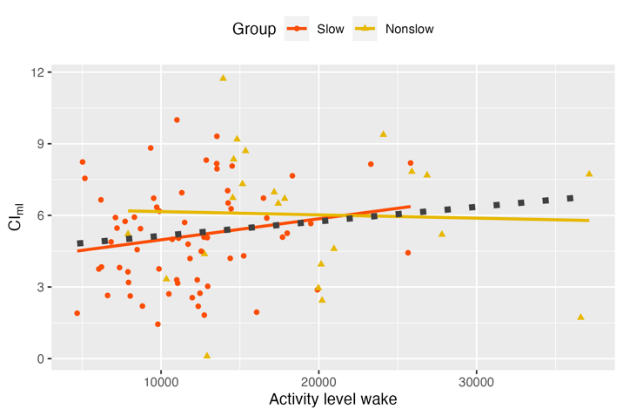
*
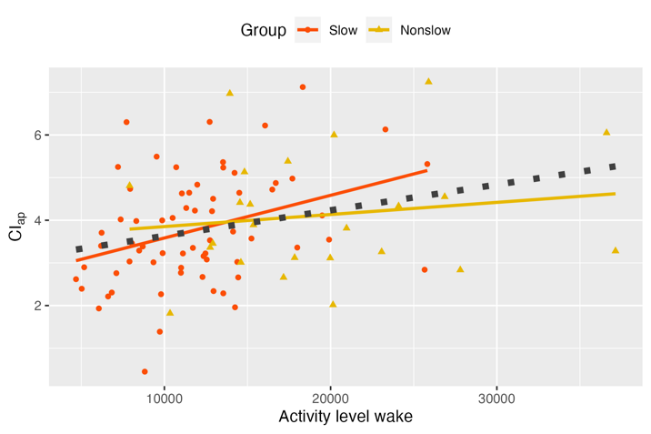


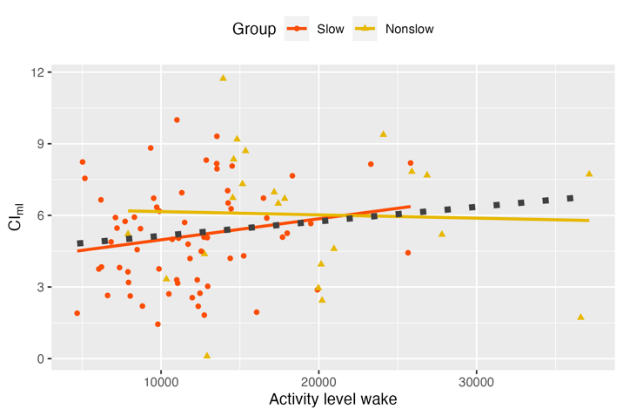

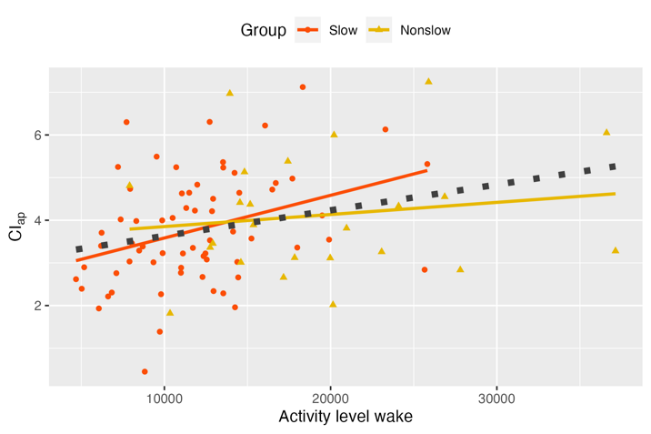

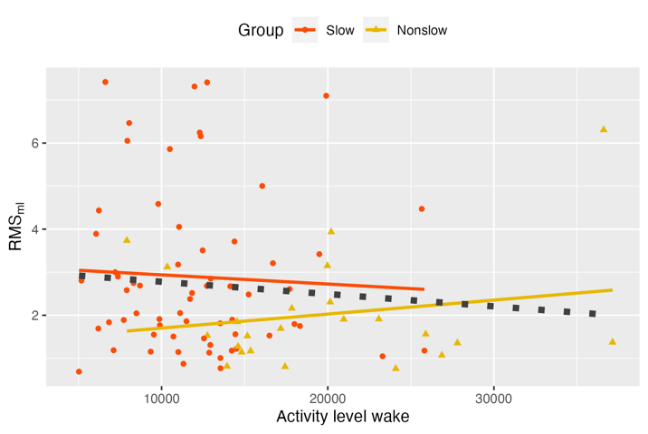


*
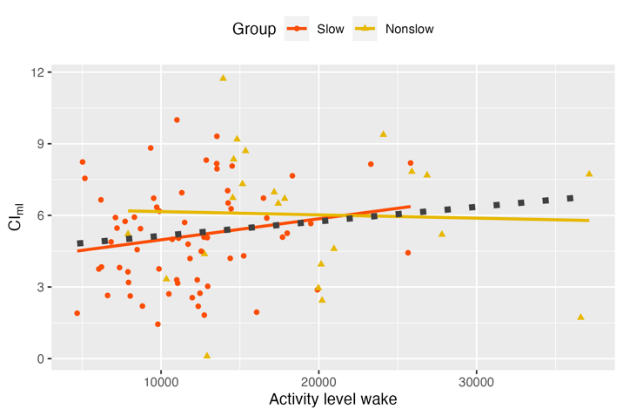
*


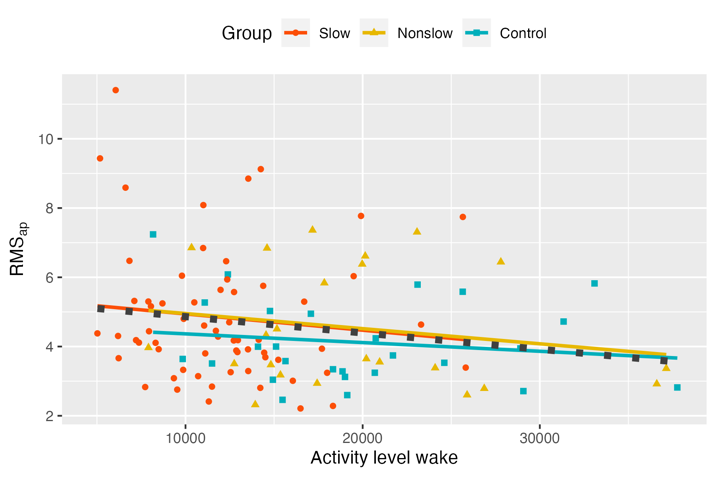

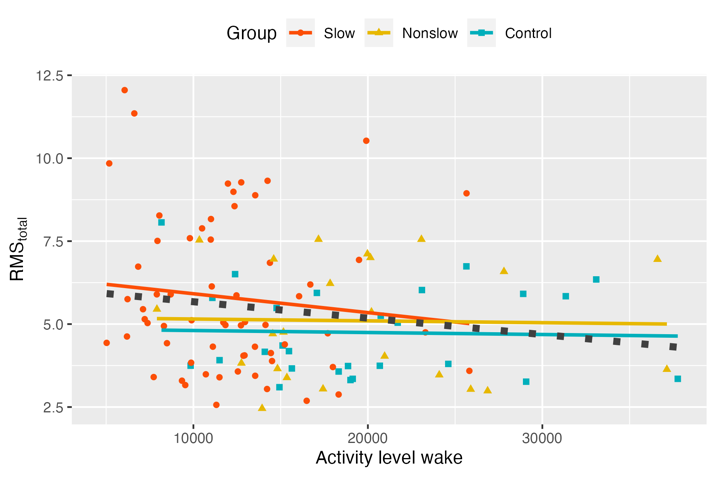

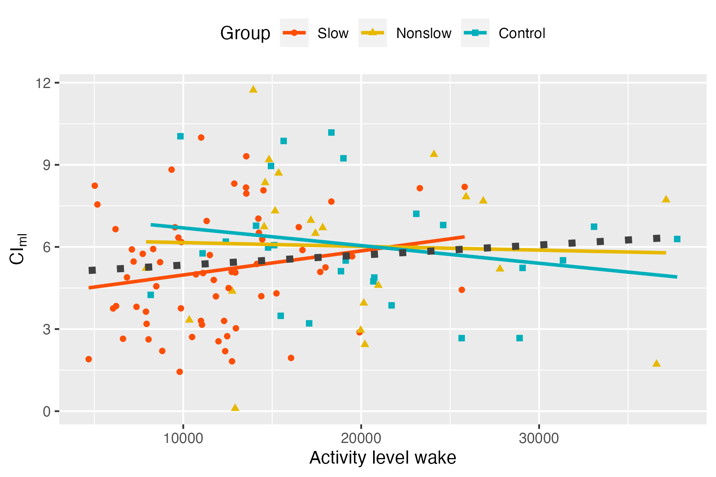


*Note*. red: PS, yellow: non-PS, blue: HC, dottet line: all respective groups included. RMS_ml_ = Root Mean Square medio-lateral; RMS_ap_ = Root Mean Square antero-posterior; RMS_total_ = Root Mean Square for total deviation; Cl_ml_ = Complexity Index medio-lateral; Cl_ap_ = Complexity Index antero-posterior.

# Associations between sway parameters and complexity

**Methods**

Associations between swaying parameters (RMS, sway area) and complexity were assessed using Kendall’s tau. These associations were calculated across all participants collectively (Table S14), as well as within the psychomotor slow (PS) group for each condition (Table S15). In the figure, the overall linear regression line is depicted, along with the individual slopes for each group (Figure S5). Only the EO condition is illustrated in the figure.

**Results**

The associations between swaying parameters and complexity were consistently negative. This indicates that greater swaying, as reflected by higher RMS and sway area, corresponds to lower complexity. These associations were significant also only within the PS group, suggesting that they are not artifacts caused by differences between the groups.

Table S14. Kendall’s Tau for swaying versus complexity per condition with all participants (N = 125).

|  |  | RMS_ml_ | | RMS_ap_ | | RMS_total_ | | Sway area | |
| --- | --- | --- | --- | --- | --- | --- | --- | --- | --- |
|  |  | Tau | p | Tau | p | Tau | p | Tau | p |
| EO | CI_ml_ | -0.669 | <.001* | -0.196 | .001* | -0.383 | <.001* | -0.419 | <.001* |
|  | CI_ap_ | -0.104 | 0.09 | -0.513 | <.001* | -0.409 | <.001* | -0.211 | <.001* |
|  |  |  |  |  |  |  |  |  |  |
| EC | CI_ml_ | -0.629 | < .001* | -0.340 | <.001* | -0.427 | <.001* | -0.504 | <.001* |
|  | CI_ap_ | -0.113 | .068 | -0.436 | <.001* | -0.385 | <.001* | -0.217 | <.001* |
|  |  |  |  |  |  |  |  |  |  |
| EOHR | CI_ml_ | -0.658 | < .001* | -0.310 | <.001* | -0.420 | <.001* | -0.487 | <.001* |
|  | CI_ap_ | -0.134 | .029* | -0.520 | <.001* | -0.466 | <.001* | -0.253 | <.001* |
|  |  |  |  |  |  |  |  |  |  |
| ECHR | CI_ml_ | -0.555 | < .001* | -0.190 | .002* | -0.276 | <.001* | -0.366 | <.001* |
|  | CI_ap_ | -0.015 | .809 | -0.410 | <.001* | -0.364 | <.001* | -0.121 | .050* |

*Note*. RMS_ml_ = Root Mean Square medio-lateral; RMS_ap_ = Root Mean Square antero-posterior; RMS_total_ = Root Mean Square for total deviation; Cl_ml_ = Complexity Index medio-lateral; Cl_ap_ = Complexity Index antero-posterior; EO = eyes open, natural upright head position, and feet hip-width apart; EC = eyes closed natural upright head position, and feet hip-width apart; EOHR = eyes open, head reclined, and feet hip-width apart; ECHR = eyes closed, head reclined, and feet hip-width apart.
* p < 0.05

Table S15. Kendall’s Tau for swaying versus complexity per condition with only PS (N = 73).

|  |  | RMS_ml_ | | RMS_ap_ | | RMS_total_ | | Sway area | |
| --- | --- | --- | --- | --- | --- | --- | --- | --- | --- |
|  |  | Tau | p | Tau | p | Tau | p | Tau | p |
| EO | CI_ml_ | -0.653 | <.001* | -0.233 | .004* | -0.391 | <.001* | -0.426 | <.001* |
|  | CI_ap_ | -0.072 | .375 | -0.434 | <.001* | -0.349 | <.001* | -0.163 | .049* |
|  |  |  |  |  |  |  |  |  |  |
| EC | CI_ml_ | -0.591 | <.001* | -0.252 | .002* | -0.355 | <.001* | -0.442 | <.001* |
|  | CI_ap_ | -0.066 | .420 | -0.343 | <.001* | -0.304 | <.001* | -0.179 | .026* |
|  |  |  |  |  |  |  |  |  |  |
| EOHR | CI_ml_ | -0.653 | <.001* | -0.333 | <.001* | -0.446 | <.001* | -0.454 | <.001* |
|  | CI_ap_ | -0.138 | .087 | -0.483 | <.001* | -0.426 | <.001* | -0.221 | .006* |
|  |  |  |  |  |  |  |  |  |  |
| ECHR | CI_ml_ | -0.531 | <.001* | -0.203 | .012* | -0.279 | <.001* | -0.312 | <.001* |
|  | CI_ap_ | -0.049 | .542 | -0.364 | <.001* | -0.313 | <.001* | -0.137 | .095 |

*Note*. RMS_ml_ = Root Mean Square medio-lateral; RMS_ap_ = Root Mean Square antero-posterior; RMS_total_ = Root Mean Square for total deviation; Cl_ml_ = Complexity Index medio-lateral; Cl_ap_ = Complexity Index antero-posterior; EO = eyes open, natural upright head position, and feet hip-width apart; EC = eyes closed natural upright head position, and feet hip-width apart; EOHR = eyes open, head reclined, and feet hip-width apart; ECHR = eyes closed, head reclined, and feet hip-width apart.
* p < 0.05

Figure S5. Scatterplot and linear regression line of swaying versus complexity in EO condition.


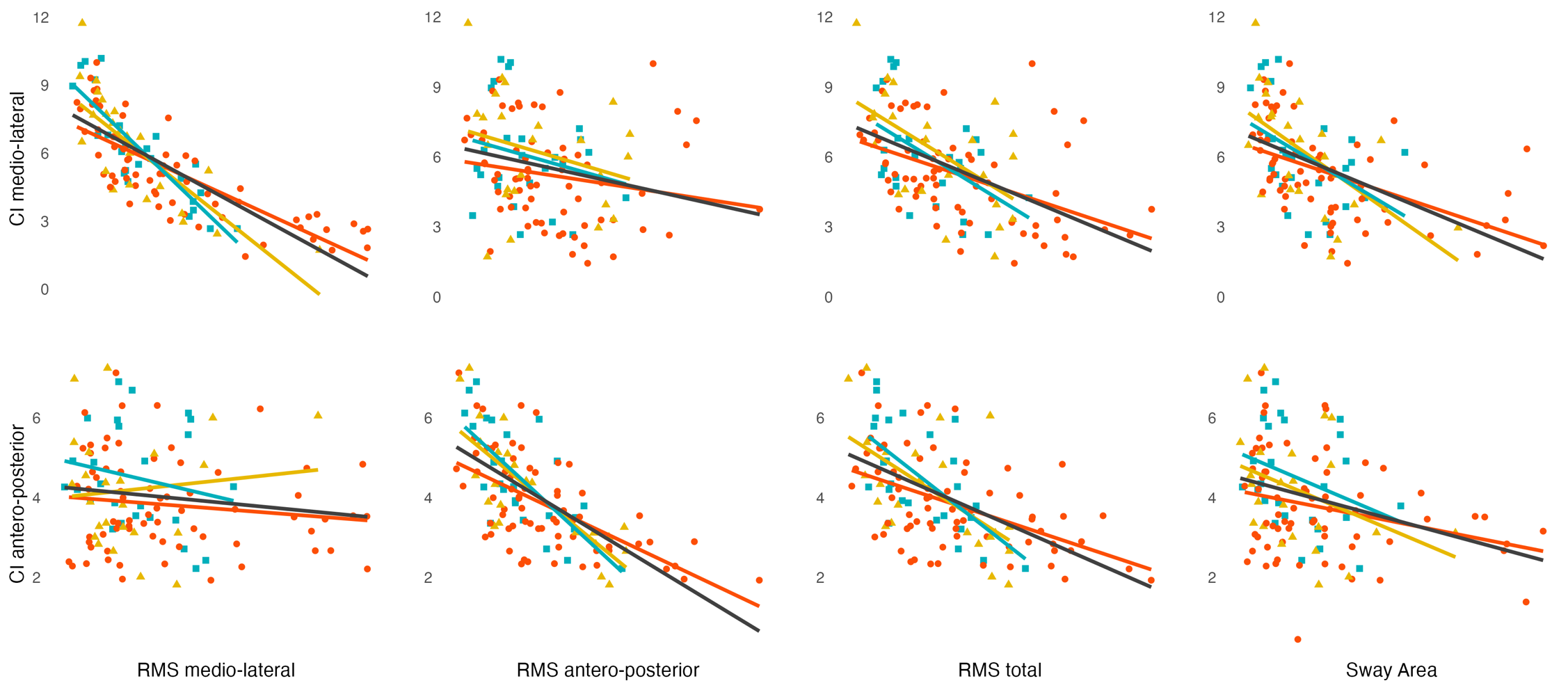


*Note*. CI = Complexity Index, TMS = Root Mean Square, red circles = PS, yellow triangles = non-PS, blue squares = HC, black line = all together.

# Analyses including outliers

**Methods**

In this study, we identified outliers for each condition separately for all six parameters. If one participant was an outlier in one of the three RMS measures, all RMS measures were excluded.

For RMS, 10 participants were classified as outliers in at least one condition, including 2 healthy controls (HC), 2 non-psychomotor slowed (non-PS) participants, and 6 psychomotor slowed (PS) participants, with one PS participant being an outlier in two conditions. For sway area, 12 participants were classified as outliers in at least one condition: 2 HC (one in two conditions), 3 non-PS, and 7 PS participants (one in two conditions and one in three conditions). The complexity had no outliers. For one participant the complexity was not included for conditions with the head reclined (EOHR, ECHR), as their data was an outlier everywhere else with the head reclined. We suspected that there might have something been wrong during the measurement with the head reclined.

Overall, 9 PS participants, 3 non-PS participants, and 2 HC were classified as outliers in at least one condition and one parameter. We further examined the demographics of these participants (Table S16) and then repeated the statistical analyses that are reported main paper with these participants included (Table S17, Figure S6, Table S18).

**Results**

On average, the outliers were slightly older than the included sample, with similar BMI, education levels, and medication use. Possibly due to the higher age, the duration of illness was also higher among outliers, though the number of episodes remained similar. Severity of psychopathology, including PANSS and BNSS scores, were comparable across groups. PS participants showed slightly higher levels of parkinsonism and neurological soft signs, but similar other motor abnormalities were observed across groups (SRRS, BFCRS). When looking at the violin plot, it is clearly visible that the outliers are not just on the end of the spectrum, but sometimes incredibly far from the rest of the group. It does seem sensible to exclude these participants.

The results of the ANOVA change slightly with the outliers included, but there are still similar main effects: there is a group difference in every parameter, condition difference in almost every parameter, and no interactions. There are fewer significant post-hoc tests, but the overall picture remains the same. Overall, we see most differences between PS and HC. There are differences between PS and non-PS when looking at the amount of swaying (RMS, sway area). There are differences between non-PS and HC when looking at the complexity. So, the conclusion, that psychomotor slowing has a greater effect on the amount of swaying than on the complexity and a schizophrenia diagnosis alone does already reduce complexity (without the addition of psychomotor slowing), is still valid after including the outliers. The associations including the outliers remain similar to analyses without the outliers. Some correlations increase slightly in strength, most strongly in the NES.

Table S16. Demographic and clinical characteristics of excluded outliers.

|  | PS | non-PS | HC |
| --- | --- | --- | --- |
| N | 9 | 3 | 2 |
| Sex (N/%) female | 3 / 33% | 2 / 66% | 1 / 50% |
|  | mean ± sd | mean ± sd | mean ± sd |
| Age (years) | 46.1 ± 15.6 | 41.7 ± 17.2 | 38.5 ± 24.7 |
| BMI | 25.1 ± 5.0 | 27.0 ± 7.6 | 20.3 ± 0.6 |
| Education (years) | 13.8 ± 3.5 | 12.7 ± 0.6 | 14.8 ± 0.6 |
| Duration of illness (years) | 17.6 ± 17.1 | 20.7 ± 23.3 |  |
| Number of episodes | 3.8 ± 2.9 | 20.7 ± 31.5 |  |
| OLZ eq. | 14.3 ± 9.6 | 21.9 ± 16.6 |  |
| SRRS | 24.1 ± 8.0 | 9.3 ± 3.5 | 1.0 ± 1.4 |
| mSRRS | 12.4 ± 3.0 | 3.0 ± 1.0 | 0.0 ± 0.0 |
| UPDRS | 30.1 ± 14.1 | 9.7 ± 4.6 | 1.0 ± 1.4 |
| BFCRS | 8.1 ± 6.2 | 0.7 ± 0.6 | 0.0 ± 0.0 |
| NES total | 25.7 ± 14.9 | 13.0 ± 3.5 |  |
| sensory integration | 4.9 ± 3.1 | 2.0 ± 1.7 |  |
| motor coordination | 3.8 ± 3.6 | 1.3 ± 0.6 |  |
| sequencing | 7.8 ± 4.1 | 4.0 ± 4.4 |  |
| others | 9.2 ± 6.7 | 5.7 ± 4.6 |  |
| Activity level | 12113 ± 7852 | 15376 ± 3479 | 22739 ± 14636 |
| PANSS total | 82.7 ± 18.7 | 73.0 ± 11.4 |  |
| positive | 14.7 ± 6.8 | 22.0 ± 6.1 |  |
| negative | 26.4 ± 8.6 | 15.0 ± 3.6 |  |
| general | 41.6 ± 7.9 | 36.0 ± 7.5 |  |
| BNSS total | 45.4 ± 13.4 | 19.0 ± 15.6 |  |
| Anhedonia | 11.9 ± 4.8 | 4.7 ± 5.7 |  |
| Distress | 2.9 ± 2.3 | 1.3 ± 2.3 |  |
| Asocial | 7.7 ± 2.4 | 3.7 ± 2.3 |  |
| Avolition | 6.1 ± 4.0 | 5.7 ± 2.5 |  |
| Affect | 12.6 ± 4.1 | 3.3 ± 4.2 |  |
| Alogia | 4.3 ± 3.7 | 0.3 ± 0.6 |  |

*Note*. PS = patients with psychomotor slowing; non-PS = patients without psychomotor slowing; HC = healthy controls; N = number of participants; sd = standard deviation; BMI = Body Mass Index; OLZ eq. = olanzapine equivalent in mg/day; SRRS = Salpêtrière Retardation Rating Scale; mSRRS = motor part of the SRRS; UPDRS = Unified Parkinson Disease Rating Scale Part III; BFCRS = Bush-Francis Catatonia Rating Scale; NES = Neurological Evaluation Scale; PANSS = Positive And Negative Syndrom Scale; BNSS = Brief Negative Symptom Scale.

* p < 0.05


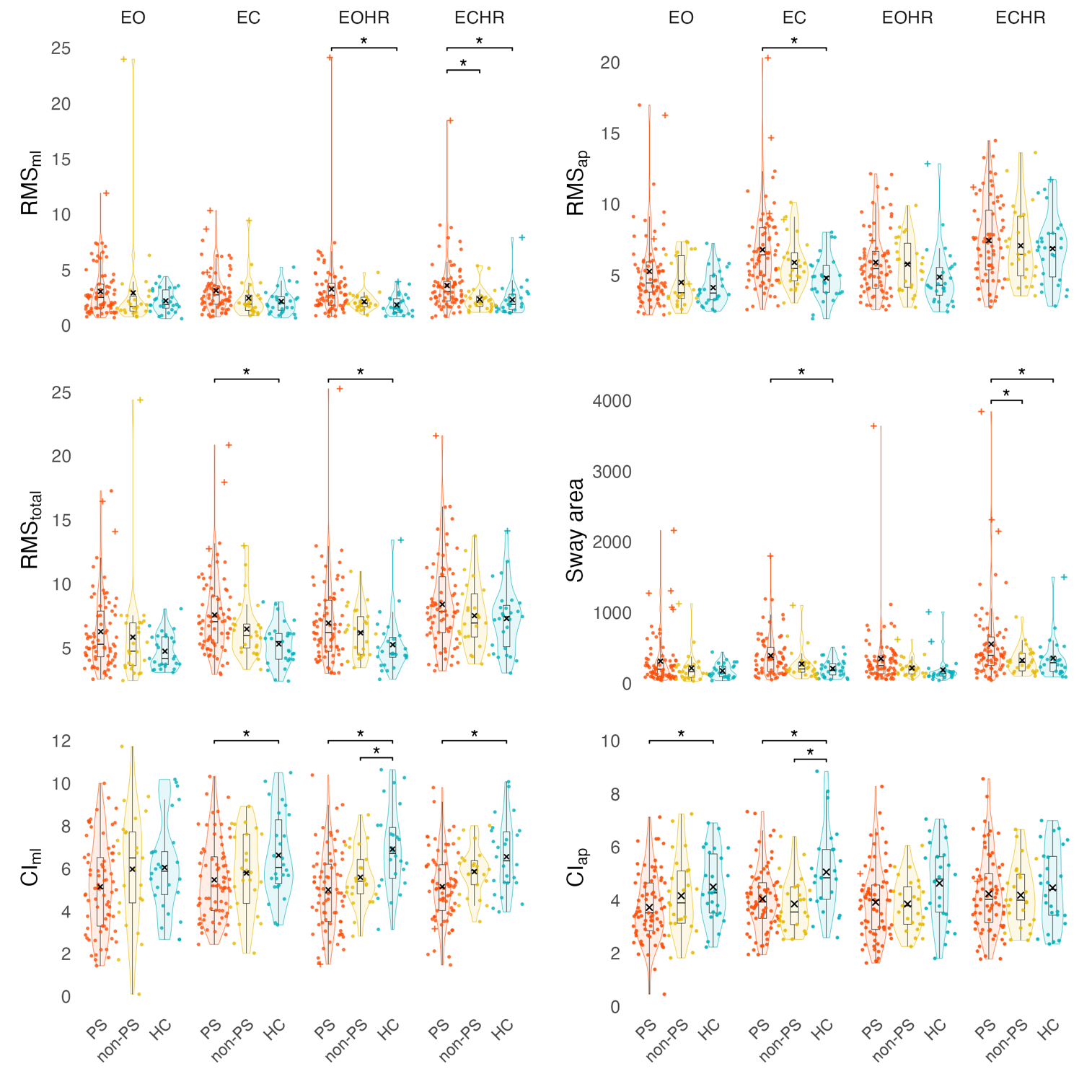
Figure S6. Differences between groups per condition for postural parameters including outliers

*Note*. Points are individuals that are included in the main analysis. Plus (+) shape represent participants that were removed in the main analysis and are additionally included here (outliers). Violin shows the approximate distribution, boxplot the quartiles (1st, median, 3rd), black x indicates the mean. red = PS, yellow = non-PS, blue = HC. EO = eyes open, natural upright head position, and feet hip-width apart ; EC = eyes closed natural upright head position, and feet hip-width apart; EOHR = eyes open, head reclined, and feet hip-width apart; ECHR = eyes closed, head reclined, and feet hip-width apart; PS = patients with psychomotor slowing; non-PS = patients without psychomotor slowing; HC = healthy controls; RMS_ml_ = Root Mean Square medio-lateral; RMS_ap_ = Root Mean Square antero-posterior; RMS_total_ = Root Mean Square for total deviation; Cl_ml_ = Complexity index medio-lateral; Cl_ap_ = Complexity index antero-posterior

* p < 0.05

Table S17. Effect of group and condition on postural stability for each posture parameter and post-hocs for groups including outliers.

|  | Main ANOVAs | | | |  |  | PS |  | non-PS |  | HC |  | p-value for posthocs btw groups | | |
| --- | --- | --- | --- | --- | --- | --- | --- | --- | --- | --- | --- | --- | --- | --- | --- |
|  |  | F | numDF, denDF | p | Conditions |  | mean ± sd | N | mean ± sd | N | mean ± sd | N | PS vs. HC | PS vs. non-PS | non-PS  vs HC |
| RMS_ml_ | Group  Condition Group*Condition | 5.08  1.24  1.13 | 2,122  3,365  6,365 | .008*  .295  .347 |  | EO  EC  EOHR  ECHR | 3.04 ± 2.12  3.12 ± 1.88  3.26 ± 2.92  3.59 ± 2.52 | 73  73  73  72 | 2.92 ± 4.56  2.45 ± 1.83  2.13 ± 0.75  2.35 ± 1.05 | 25  25  25  25 | 2.20 ± 1.02  2.15 ± 1.08  1.84 ± 0.85  2.29 ± 1.39 | 27  27  27  27 | .217  .128  .014*  .023* | .970  .390  .074  .036* | .475  .877  .881  .996 |
| RMS_ap_ | Group  Condition Group*Condition | 4.84  33.30  1.13 | 2,122  3,365  6,365 | .010*  <.001*  .346 |  | EO  EC  EOHR  ECHR | 5.27 ± 2.65  6.79 ± 2.82  5.90 ± 2.34  7.438 ± 2.81 | 73  73  73  72 | 4.49 ± 1.65  5.89 ± 1.81  5.77 ± 1.87  7.07 ± 2.63 | 25  25  25  25 | 4.12 ± 1.23  4.79 ± 1.55  4.86 ± 2.15  6.87 ± 2.36 | 27  27  27  27 | .090  .001*  .136  .547 | .346  .245  .971  .786 | .845  .227  .361  .951 |
| RMS_total_ | Group  Condition Group*Condition | 5.73  20.58  0.558 | 2,122  3,365  6,365 | .004*  <.001  .764 |  | EO  EC  EOHR  ECHR | 6.28 ± 3.01  7.58 ± 3.14  6.95 ± 3.34  8.41 ± 3.42 | 73  73  73  72 | 5.85 ± 4.22  6.48 ± 2.29  6.19 ± 1.90  7.53 ± 2.62 | 25  25  25  25 | 4.75 ± 1.35  5.32 ± 1.68  5.26 ± 2.16  7.31 ± 2.58 | 27  27  27  27 | .058  .003*  .032*  .205 | .803  .246  .506  .376 | .372  .331  .494  .960 |
| Sway Area | Group  Condition Group*Condition | 4.15  19.96  0.75 | 2,121  3,361  6,361 | .018*  <.001*  .606 |  | EO  EC  EOHR  ECHR | 313.2 ± 356.5  391.2 ± 310.5  347.8 ± 446.0  554.2 ± 582.4 | 73  72  73  72 | 215.2 ± 226.1  273.5 ± 223.4  217.0 ± 123.6  325.0 ± 186.3 | 25  25  25  25 | 177.8 ± 101.6  209.6 ± 124.1  189.5 ± 196.6  356.1 ± 288.9 | 26  26  26  26 | .248  .038*  .151  .047* | .489  .209  .282  .020* | .931  .811  .962  .952 |
| CI_ml_ | Group  Condition Group*Condition | 7.97  1.16  1.43 | 2,121  3,362  6,362 | .001*  .324  .201 |  | EO  EC  EOHR  ECHR | 5.14 ± 2.10  5.47 ± 1.80  5.00 ± 1.87  5.15 ± 1.64 | 73  73  73  72 | 5.97 ± 2.70  5.80 ± 1.92  5.59 ± 1.32  5.85 ± 1.14 | 25  25  25  25 | 6.05 ± 2.19  6.62 ± 1.99  6.91 ± 1.92  6.55 ± 1.70 | 26  26  26  26 | .092  .022*  <.001*  .004* | .144  .742  .372  .227 | .987  .258  .036*  .381 |
| CI_ap_ | Group  Condition Group*Condition | 4.49  2.41  1.95 | 2,121  3,362  6,362 | .013*  .067  .072 |  | EO  EC  EOHR  ECHR | 3.72 ± 1.27  4.04 ± 1.19  3.92 ± 1.41  4.23 ± 1.45 | 73  73  73  72 | 4.16 ± 1.43  3.85 ± 1.00  3.85 ± 0.98  4.18 ± 1.18 | 25  25  25  25 | 4.49 ± 1.31  5.05 ± 1.56  4.63 ± 1.44  4.46 ± 1.49 | 26  26  26  26 | .033*  .003*  .051  .721 | .334  .811  .975  .989 | .645  .005*  .093  .735 |

*Note*. RMS_ml_ = Root Mean Square medio-lateral; RMS_ap_ = Root Mean Square antero-posterior; RMS_total_ = Root Mean Square for total deviation; Cl_ml_ = Complexity Index medio-lateral; Cl_ap_ = Complexity Index antero-posterior; PS = patients with psychomotor slowing; non-PS = patients without psychomotor slowing; HC = healthy controls; EO = eyes open, natural upright head position, and feet hip-width apart; EC = eyes closed natural upright head position, and feet hip-width apart; EOHR = eyes open, head reclined, and feet hip-width apart; ECHR = eyes closed, head reclined, and feet hip-width apart; N = Number of participants for the specific group and condition; sd = standard deviation; btw = between.

* p < 0.05

Table S18. Associations with outliers in EO condition

|  | RMS_ml_ | | | RMS_ap_ | | | RMS_total_ | | | Sway Area | | | CI_ml_ | | | CI_ap_ | | |
| --- | --- | --- | --- | --- | --- | --- | --- | --- | --- | --- | --- | --- | --- | --- | --- | --- | --- | --- |
|  | N | Tau | p | N | Tau | p | N | Tau | p | N | Tau | p | N | Tau | p | N | Tau | p |
| mSRRS | 98 | .191 | .007* | 98 | .175 | .013* | 98 | .191 | .007* | 98 | .161 | .023* | 98 | -.193 | .006* | 98 | -.195 | .006* |
| SRRS | 98 | .153 | .028* | 98 | .127 | .067 | 98 | .146 | .036* | 98 | .117 | .094 | 98 | -.158 | .023* | 98 | -.159 | .022* |
| UPDRS | 98 | .201 | .004* | 98 | .224 | .001* | 98 | .237 | .001* | 98 | .215 | .002* | 98 | -.178 | .010* | 98 | -.167 | .016* |
| BFCRS | 98 | .208 | .004* | 98 | .114 | .113 | 98 | .152 | .034* | 98 | .145 | .044* | 98 | -.205 | .004* | 98 | -.073 | .310 |
| NES total | 95 | .194 | .006* | 95 | .237 | .001* | 95 | .270 | <.001* | 95 | .240 | .001* | 95 | -.165 | .020* | 95 | -.108 | .128 |
| NES sensory integration | 95 | .189 | .011* | 95 | .168 | .024* | 95 | .200 | .007* | 95 | .234 | .002* | 95 | -.105 | .157 | 95 | .005 | .945 |
| NES motor coordination | 96 | .253 | .001* | 96 | .207 | .006* | 96 | .265 | <.001* | 96 | .250 | .001* | 96 | -.220 | .004* | 96 | -.110 | .145 |
| NES sequencing | 95 | .141 | .051 | 95 | .183 | .012* | 95 | .236 | .001* | 95 | .192 | .008* | 95 | -.097 | .180 | 95 | -.040 | .582 |
| NES others | 95 | .141 | .050* | 95 | .210 | .004* | 95 | .209 | .004* | 95 | .183 | .011* | 95 | -.126 | .079 | 95 | -.139 | .055 |
| Activity level | 90 | -.166 | .020* | 90 | -.163 | .023* | 90 | -.156 | .029* | 90 | -.128 | .074 | 90 | .146 | .041* | 90 | .200 | .005* |
| BNSS total | 98 | .235 | .001* | 98 | .154 | .026* | 98 | .166 | .016* | 98 | .180 | .009* | 98 | -.249 | <.001* | 98 | -.148 | .032* |
| BNSS Anhedonia | 98 | .185 | .009* | 98 | .109 | .121 | 98 | .111 | .116 | 98 | .137 | .052 | 98 | -.203 | .004* | 98 | -.079 | .260 |
| BNSS Distress | 98 | .170 | .021* | 98 | .112 | .130 | 98 | .106 | .150 | 98 | .129 | .080 | 98 | -.207 | .005* | 98 | -.119 | .105 |
| BNSS Asocial | 98 | .172 | .017* | 98 | .100 | .165 | 98 | .115 | .112 | 98 | .147 | .041* | 98 | -.155 | .031* | 98 | -.054 | .453 |
| BNSS Avolition | 98 | .218 | .003* | 98 | .101 | .163 | 98 | .121 | .094 | 98 | .153 | .034* | 98 | -.202 | .005* | 98 | -.058 | .420 |
| BNSS Affect | 98 | .227 | .001* | 98 | .185 | .009* | 98 | .191 | .007* | 98 | .197 | .005* | 98 | -.214 | .002* | 98 | -.193 | .006* |
| BNSS Alogia | 98 | .161 | .025* | 98 | .097 | .177 | 98 | .104 | .148 | 98 | .097 | .179 | 98 | -.197 | .006* | 98 | -.115 | .109 |

*Note*. mSRRS = motor part of the SRRS; SRRS = Salpêtrière Retardation Rating Scale; UPDRS = Unified Parkinson Disease Rating Scale Part III; BFCRS = Bush-Francis Catatonia Rating Scale; NES = Neurological Evaluation Scale; BNSS = Brief Negative Symptom Scale; RMS_ml_ = Root Mean Square medio-lateral; RMS_ap_ = Root Mean Square antero-posterior; RMS_total_ = Root Mean Square for total deviation; Cl_ml_ = Complexity Index medio-lateral; Cl_ap_ = Complexity Index antero-posterior; N = Number of participants for the specific postural parameter and the clinical or motor scale.

* p < 0.05

# Analyses for additional postural conditions EOTS and ECTS

**Methods**

In our main analyses, we included conditions 1 to 4 (EO, EC, EOHR, ECHR), as the two conditions with tandem stance (EOTS, ECTS) were too difficult for the participants, which lead to an unproportionally high variance (Figure S7). These conditions are (v) eyes open, head in natural upright position, and feet in tandem stance (EOTS) and vi) eyes closed, head in natural upright position, and feet in tandem stance (ECTS).

We visually compared the frequency of safety behaviours between groups and calculated chi-square tests for EOTS and ECTS (Table S19), as well as calculated an ANOVA for EOTS and ECTS separately to see group differences (Table S20).

**Results**

Five PS had to open their eyes during eyes-closed tasks (EC, ECHR) to maintain their balance (Table S19). The tandem stance (EOTS, ECTS) seemed to be challenging for all participants, as not only patients but also HC had to open their eyes or hold on to the bar. However, the frequency of safety behaviours is significantly different between the three groups, with PS needing help approximately twice as frequent as HC. Interestingly, PS and HC differ for every postural parameter in EOTS (Table S20A), while almost no group differences are detectable in the most challenging task ECTS (Table S20B)

## Frequency of Safety Behaviours

Table S19. Absolute number and frequency of safety behaviours per group and condition

|  | Open Eyes | | | | | |  | Hold the bar | | | | | |
| --- | --- | --- | --- | --- | --- | --- | --- | --- | --- | --- | --- | --- | --- |
|  | EO | EC | EOHR | ECHR | EOTS | ECTS |  | EO | EC | EOHR | ECHR | EOTS | ECTS |
| PS | - | 2/73 = 3% | - | 2/71= 3% | - | 17/55 = 31% |  | 0 | 0 | 0 | 0 | 8/68 = 12% | 42/55 = 76% |
| non-PS | - | 0/25 | - | 0/25 | - | 4/16 = 25% |  | 0 | 0 | 0 | 0 | 1/25 = 4% | 8/16 = 50% |
| HC | - | 0/27 | - | 0/27 | - | 2/25 = 8% |  | 0 | 0 | 0 | 0 | 0/26 | 10/25 = 60% |
| Chi-squared | - | - | - | - | - | X^2^ = 17.3, p < .001* |  | - | - | - | - | X^2^ = 12.7, p = .002* | X^2^ = 36.4, p < .001* |

*Note*. PS = patients with psychomotor slowing; non-PS = patients without psychomotor slowing; HC = healthy controls; EO = eyes open, natural upright head position, and feet hip-width apart; EC = eyes closed natural upright head position, and feet hip-width apart; EOHR = eyes open, head reclined, and feet hip-width apart; ECHR = eyes closed, head reclined, and feet hip-width apart; EOTS = eyes open, tandem stance; ECTS = eyes closed, tandem stance.

* p < 0.05

## Scatterplot and density plot per group for EOTS and ECTS.

Figure S7. Scatterplot and density plot for EOTS and ECTS

PS non-PS HC

EOTS

ECTS

*Note*. Same x- and y-scale is used for all plots to increase comparability. Scatterplot of all CoP measuring points of all individuals of the three groups. Density plots illustrating the dispersion and height of the scatterplots. Row 1 and 2: EOTS, row 3 and 4: ECTS. EOTS = eyes open, tandem stance; ECTS = eyes closed, tandem stance; PS = patients with psychomotor slowing; non-PS = patients without psychomotor slowing; HC = healthy controls.

.

## ANOVA and Posthocs for EOTS and ECTS separately between groups

Table S20. ANOVAs only for EOTS and ECTS separately with posthocs between groups

| 1. **EOTS** |  | | |  | | | | | |  | | |
| --- | --- | --- | --- | --- | --- | --- | --- | --- | --- | --- | --- | --- |
|  | Main effect of group | | | PS |  | non-PS |  | HC |  | p-value for posthocs between groups | | |
|  | F | numDF, denDF | p | mean ± sd | N | mean ± sd | N | mean ± sd | N | PS vs. HC | PS vs. non-PS | non-PS vs. HC |
| RMS_ml_ | 5.10 | 2,109 | .008* | 5.39 ± 1.94 | 64 | 4.98 ± 1.32 | 22 | 4.15 ± 1.13 | 26 | .005* | .586 | .202 |
| RMS_ap_ | 6.17 | 2,109 | .003* | 6.27 ± 3.22 | 64 | 4.44 ± 1.82 | 22 | 4.46 ± 1.84 | 26 | .013* | .020* | .999 |
| RMS_total_ | 6.28 | 2,109 | .003* | 8.39 ± 3.49 | 64 | 6.77 ± 1.95 | 22 | 6.16 ± 1.92 | 26 | .004* | .070 | .759 |
| Sway Area | 4.59 | 2,110 | .012* | 792.94 ± 731.32 | 64 | 546.98 ± 263.19 | 24 | 407.64 ± 201.93 | 25 | .015* | .178 | .673 |
| CI_ml_ | 6.10 | 2,116 | .003* | 5.66 ± 1.50 | 68 | 5.93 ± 1.61 | 25 | 6.89 ± 1.49 | 26 | .002* | .739 | .067 |
| CI_ap_ | 5.93 | 2,116 | .004* | 5.83 ± 1.96 | 68 | 6.40 ± 2.15 | 25 | 7.41 ± 1.99 | 26 | .002* | .445 | .172 |
| 1. **ECTS** |  |  |  |  |  |  |  |  |  |  |  |  |
|  | Main effect of group | | | PS |  | non-PS |  | HC |  | p-value for posthocs between groups | | |
|  | F | numDF, denDF | p | mean ± sd | N | mean ± sd | N | mean ± sd | N | PS vs. HC | PS vs. non-PS | non-PS vs. HC |
| RMS_ml_ | 0.54 | 2,91 | .586 | 12.64 ± 3.04 | 54 | 11.72 ± 2.78 | 16 | 12.38 ± 3.45 | 24 | .940 | .557 | .789 |
| RMS_ap_ | 2.59 | 2,91 | .080 | 16.73 ± 9.28 | 54 | 12.38 ± 6.22 | 16 | 13.00 ± 7.44 | 24 | .174 | .170 | .971 |
| RMS_total_ | 2.14 | 2,91 | .123 | 21.37 ± 8.83 | 54 | 17.45 ± 5.63 | 16 | 18.24 ± 7.52 | 24 | .259 | .208 | .951 |
| Sway Area | 1.45 | 2,91 | .241 | 5075.25 ± 3876.62 | 53 | 3354.53 ± 1905.92 | 16 | 4379.52 ± 3876.13 | 25 | .710 | .225 | .652 |
| CI_ml_ | 3.20 | 2,93 | .045* | 5.58 ± 1.42 | 55 | 5.85 ± 1.36 | 16 | 6.42 ± 1.29 | 25 | .035* | .772 | .401 |
| CI_ap_ | 1.26 | 2,93 | .290 | 5.20 ± 2.08 | 55 | 5.68 ± 2.09 | 16 | 5.96 ± 2.05 | 25 | .284 | .691 | .908 |

*Note*. Posthocs are Tukey corrected for multiple comparison within each model (3 comparisons). Outliers are excluded per condition and per postural parameter separately leading to different sample sizes. RMS_ml_ = Root Mean Square medio-lateral; RMS_ap_ = Root Mean Square antero-posterior; RMS_total_ = Root Mean Square for total deviation; Cl_ml_ = Complexity Index medio-lateral; Cl_ap_ = Complexity Index antero-posterior; PS = patients with psychomotor slowing; non-PS = patients without psychomotor slowing; HC = healthy controls; EOTS = eyes open, tandem stance; ECTS = eyes closed, tandem stance; N = Number of participants for the specific postural parameter and group; sd = standard deviation.

* p < 0.05

# ANOVA and Posthocs for Sample Entropy over several timescales between groups

**Methods**

*Calculation of Sample Entropy, Multiscale Sample Entropy, and the Complexity Index*

- - 1. **Sample Entropy Calculation**

Sample Entropy is a method used to quantify the unpredictability or irregularity of time-series data. It evaluates how often patterns of data repeat over time. Sample Entropy is particularly useful in assessing physiological signals, such as center of pressure (CoP) in postural sway, where more irregular signals may indicate more adaptability or complexity in the system.

The calculation involves several steps:

1. **Sequence length (m):** Choose a pattern length *m*, which defines how many consecutive data points are compared as a sequence. For example, if *m = 2*, the algorithm looks at pairs of consecutive data points. We chose *m = 2*.
2. **Tolerance (r):** Define a threshold *r* within which two sequences are considered "similar." Typically, *r* is a percentage of the standard deviation of the time series (e.g., 15%). We chose *r* = 15.
3. **Match Count (A and B):**
   1. Construct sequences of length *m* and *m+1* from the time series data.
   2. Calculate the maximum difference between these sequences.
   3. Count how many sequences of length *m* are similar (difference less than *r*) and store this as *B*.
   4. Count how many sequences of length *m+1* are similar and store this as *A*.
4.
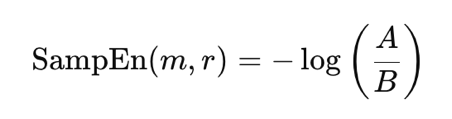
Entropy Calculation: Sample Entropy is defined as the negative natural logarithm of the ratio of the number of similar sequences of length *m+1* (*A*) to the number of similar sequences of length *m* (*B*):
   - 1. **Multiscale Sample Entropy (MSE) Calculation**

Multiscale Sample Entropy is an extension of Sample Entropy that evaluates the complexity of time-series data across multiple temporal scales, rather than at just one scale. This method allows researchers to assess both short-term and long-term patterns in the data.

The steps for calculating MSE are as follows:

1. **Coarse-Graining the Time Series:** For each time scale *τ*, the original time series is coarse-grained by averaging consecutive data points. For example:
   - At *τ = 1*, the original time series is used.
   - At *τ = 2*, every two consecutive data points are averaged to create a new time series.
   - At *τ = 3*, every three consecutive data points are averaged, and so on.

This process effectively reduces the resolution of the time series, allowing for the examination of slower, long-term fluctuations. For each timescale 200 datapoints should be available for analysis. We chose to calculate the MSE using 6 timescales*, as we had 1200 original datapoints per measurement, which leaves 200 datapoints when τ = 6.*

1. Compute Sample Entropy at each timescale:
   - For each coarse-grained time series, calculate the Sample Entropy using the same *m* and r parameters as in the single-scale Semple Entropy calculation.
   - This provides an entropy value for each time scale, offering insights into the complexity of the signal at both short (small *τ*) and long (large *τ*) time scales.
     1. **Complexity Index (CI) Calculation**

The Complexity Index is a single value that summarizes the overall complexity of a system across multiple time scales. It is calculated by integrating the Sample Entropy values across the chosen time scales. In other words, the CI represents the area under the curve (AUC) when plotting Sample Entropy versus the time scale τ.


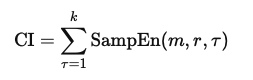


**Key Parameters**:

- **Sequence length (m):** Sequence length for comparison (usually 2 in physiological studies).
- **Tolerance (r):** Similarity threshold (often 15% of the standard deviation).
- **Time scales (τ):** Number of scales used for coarse-graining (6 in our case).

The analysis was implemented in MATLAB using publicly available code (John, M. *MATLAB Central File Exchange*. <https://www.mathworks.com/matlabcentral/fileexchange/62706-multiscale-sample-entropy> (2024)).

**Results**

The Sample Entropy showed a group difference for timescale 1 to 6 in the medio-lateral direction and for timescale 1 to 3 in the ap-direction (Table S21 and S22, Figure S8).

Figure S8. Sample Entropy over 6 timescales in medio-lateral and antero-posterior direction per group and condition


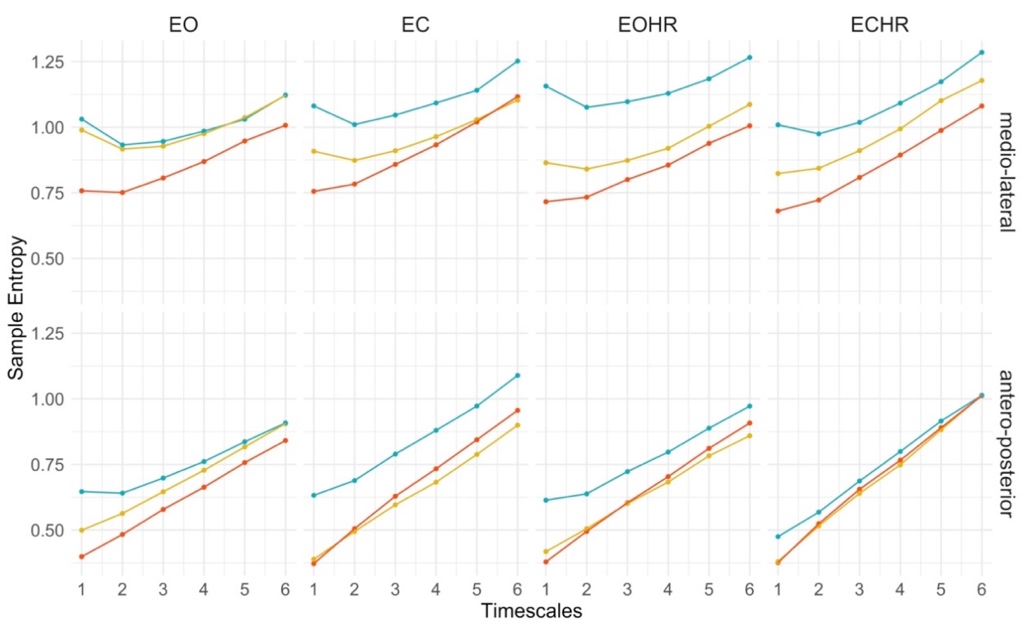


*Note*. red: PS, yellow: non-PS, blue: HC. EO = eyes open, natural upright head position, and feet hip-width apart; EC = eyes closed natural upright head position, and feet hip-width apart; EOHR = eyes open, head reclined, and feet hip-width apart; ECHR = eyes closed, head reclined, and feet hip-width apart.

Table S21. Effect of group and condition on postural stability for Sample Entropy medio-lateral 1-6 and post-hocs for groups

|  | Main ANOVAs | | | |  |  | PS |  | non-PS |  | HC |  |  | p-value for posthocs btw groups | | |
| --- | --- | --- | --- | --- | --- | --- | --- | --- | --- | --- | --- | --- | --- | --- | --- | --- |
|  |  | F | numDF, denDF | p | Conditions |  | mean ± sd | N | mean ± sd | N | mean ± sd | N |  | PS vs. HC | PS vs. non-PS | non-PS  vs HC |
| SE_ml1_ | Group  Condition Group*Condition | 10.44  3.52  1.37 | 2,121  3,360  6,360 | < .001*  .015*  .228 |  | EO  EC  EOHR  ECHR | 0.76 ± 0.43  0.76 ± 0.40  0.72 ± 0.40  0.68 ± 0.35 | 73  73  72  71 | 0.99 ± 0.53  0.91 ± 0.40  0.86 ± 0.29  0.82 ± 0.26 | 25  25  25  25 | 1.03 ± 0.47  1.08 ± 0.43  1.16 ± 0.40  1.01 ± 0.35 | 26  26  26  26 | Posthocs | .009*  .002*  <.001*  <.001* | .036*  .228  .222  .224 | .927  .274  .027  .225 |
| SE_ml2_ | Group  Condition Group*Condition | 8.87  1.22  1.36 | 2,121  3,360  6,360 | < .001*  .302  .228 |  | EO  EC  EOHR  ECHR | 0.75 ± 0.36  0.78 ± 0.32  0.73 ± 0.33  0.72 ± 0.28 | 73  73  72  71 | 0.92 ± 0.45  0.87 ± 0.33  0.84 ± 0.22  0.84 ± 0.19 | 25  25  25 | 0.93 ± 0.39  1.01 ± 0.36  1.08 ± 0.35  0.97 ± 0.30 | 26  26  26  26 |  | .046*  .009*  <.001*  .002* | .082  .468  .318  .218 | .984  .306  .032*  .335 |
| SE_ml3_ | Group  Condition Group*Condition | 7.29  1.17  1.32 | 2,121  3,360  6,360 | .001*  .322  .246 |  | EO  EC  EOHR  ECHR | 0.81 ± 0.34  0.86 ± 0.30  0.80 ± 0.30  0.81 ± 0.26 | 73  73  72  71 | 0.93 ± 0.42  0.91 ± 0.31  0.87 ± 0.20  0.91 ± 0.17 | 25  25  25  25 | 0.95 ± 0.37  1.05 ± 0.34  1.10 ± 0.33  1.02 ± 0.27 | 26  26  26  26 |  | .120  .022*  <.001*  .007* | .208  .743  .534  .283 | .976  .258  .028*  .424 |
| SE_ml4_ | Group  Condition Group*Condition | 6.21  1.86  1.35 | 2,121  3,360  6,360 | .003*  .136  .233 |  | EO  EC  EOHR  ECHR | 0.87 ± 0.34  0.93 ± 0.29  0.86 ± 0.29  0.89 ± 0.27 | 73  73  72  71 | 0.98 ± 0.43  0.96 ± 0.32  0.92 ± 0.20  0.99 ± 0.19 | 25  25  25  25 | 0.99 ± 0.35  1.09 ± 0.32  1.13 ± 0.32  1.09 ± 0.27 | 26  26  26  26 |  | .215  058  <.001*  .011* | .284  .898  .604  .292 | .993  .285  .039*  .479 |
| SE_ml5_ | Group  Condition Group*Condition | 4.70  2.62  1.47 | 2,121  3,360  6,360 | .011*  .051  .188 |  | EO  EC  EOHR  ECHR | 0.95 ± 0.34  1.02 ± 0.29  0.94 ± 0.29  0.99 ± 0.26 | 73  73  72  71 | 1.04 ± 0.44  1.03 ± 0.30  1.00 ± 0.26  1.10 ± 0.21 | 25  25  25  25 | 1.03 ± 0.32  1.14 ± 0.28  1.18 ± 0.32  1.17 ± 0.28 | 26  26  26  26 |  | .460  .195  .001*  .019* | .416  .992  .600  .215 | .997  .389  .088  .674 |
| SE_ml6_ | Group  Condition Group*Condition | 5.59  4.40  1.33 | 2,121  3,360  6,360 | .005*  .005*  242 |  | EO  EC  EOHR  ECHR | 1.01 ± 0.33  1.12 ± 0.28  1.01 ± 0.29  1.08 ± 0.29 | 73  73  72  71 | 1.12 ± 0.49  1.10 ± 0.33  1.09 ± 0.30  1.18 ± 0.23 | 25  25  25  25 | 1.12 ± 0.35  1.25 ± 0.32  1.27 ± 0.29  1.29 ± 0.34 | 26  26  26  26 |  | .251  .150  .001*  .013* | .268  .982  .486  .350 | .999  .217  .111  .449 |

*Note*. SE_ml_ = Sample Entropy medio-lateral; PS = patients with psychomotor slowing; non-PS = patients without psychomotor slowing; HC = healthy controls; EO = eyes open, natural upright head position, and feet hip-width apart; EC = eyes closed natural upright head position, and feet hip-width apart; EOHR = eyes open, head reclined, and feet hip-width apart; ECHR = eyes closed, head reclined, and feet hip-width apart; N = Number of participants for the specific condition and group; sd = standard deviation; btw = between.

* p < 0.05

Table S22. Effect of group and condition on postural stability for Sample Entropy antero-posterior 1-6 and post-hocs for groups

|  | Main ANOVAs | | | |  |  | PS |  | non-PS |  | HC |  |  | p-value for posthocs btw groups | | |
| --- | --- | --- | --- | --- | --- | --- | --- | --- | --- | --- | --- | --- | --- | --- | --- | --- |
|  |  | F | numDF, denDF | p | Conditions |  | mean ± sd | N | mean ± sd | N | mean ± sd | N |  | PS vs. HC | PS vs. non-PS | non-PS  vs HC |
| SE_ap1_ | Group  Condition Group*Condition | 12.26  5.40  3.08 | 2,121  3,360  6,360 | <.001*  .001*  .006* |  | EO  EC  EOHR  ECHR | 0.40 ± 0.22  0.37 ± 0.17  0.38 ± 0.19  0.38 ± 0.18 | 73  73  72  71 | 0.50 ± 0.26  0.39 ± 0.14  0.42 ± 0.23  0.38 ± 0.17 | 25  25  25  25 | 0.65 ± 0.34  0.63 ± 0.36  0.61 ± 0.30  0.48 ± 0.23 | 26  26  26  26 | Posthocs | <.001*  <.001*  <.001*  .131 | .136  .945  .729  .994 | .056  <.001*  .007*  .293 |
| SE_ap2_ | Group  Condition Group*Condition | 7.41  0.21  2.40 | 2,121  3,360  6,360 | <.001*  .887  .028 |  | EO  EC  EOHR  ECHR | 0.48 ± 0.19  0.51 ± 0.17  0.50 ± 0.20  0.52 ± 0.19 | 73  73  72  71 | 0.56 ± 0.24  0.49 ± 0.14  0.51 ± 0.17  0.52 ± 0.16 | 25  25  25  25 | 0.64 ± 0.24  0.69 ± 0.27  0.64 ± 0.23  0.57 ± 0.20 | 26  26  26  26 |  | .002*  <.001*  .006*  .590 | .191  .971  .975  .984 | .347  .002*  .047*  .614 |
| SE_ap3_ | Group  Condition Group*Condition | 4.55  2.15  2.07 | 2,121  3,360  6,360 | .013*  .094  .056 |  | EO  EC  EOHR  ECHR | 0.58 ± 0.20  0.63 ± 0.19  0.60 ± 0.22  0.66 ± 0.22 | 73  73  72  71 | 0.65 ± 0.24  0.60 ± 0.16  0.60 ± 0.15  0.64 ± 0.17 | 25  25  25  25 | 0.70 ± 0.21  0.79 ± .26  0.72 ± 0.23  0.69 ± 0.23 | 26  26  26  26 |  | .035*  .003*  .039*  .787 | .346  .778  .997  .945 | .646  .004  .097  .700 |
| SE_ap4_ | Group  Condition Group*Condition | 2.83  4.59  1.71 | 2,121  3,360  6,360 | .063  .004*  .119 |  | EO  EC  EOHR  ECHR | 0.66 ± 0.22  0.73 ± 0.21  0.70 ± 0.24  0.77 ± 0.27 | 73  73  72  71 | 0.73 ± 0.25  0.68 ± 0.18  0.68 ± 0.16  0.75 ± 0.21 | 25  25  25  25 | 0.76 ± 0.21  0.88 ± 0.26  0.80 ± 0.25  0.80 ± 0.28 | 26  26  26  26 |  | .161  .018  .196  .809 | .457  .612  .912  .943 | .866  .008*  .189  .717 |
| SE_ap5_ | Group  Condition Group*Condition | 1.53  7.52  1.31 | 2,121  3,360  6,360 | .221  <.001*  .250 |  | EO  EC  EOHR  ECHR | 0.76 ± 0.24  0.84 ± 0.24  0.81 ± 0.29  0.89 ± 0.31 | 73  73  72  71 | 0.82 ± 0.25  0.79 ± 0.20  0.78 ± 0.19  0.88 ± 0.26 | 25  25  25  25 | 0.84 ± 0.22  0.97 ± 0.26  0.89 ± 0.27  0.91 ± 0.33 | 26  26  26  26 |  | .388  .088  .430  .910 | .591  .631  .874  .990 | .962  .037*  .331  .895 |
| SE_ap6_ | Group  Condition Group*Condition | 0.95  11.32  1.53 | 2,121  3,360  6,360 | .392  <.001*  .168 |  | EO  EC  EOHR  ECHR | 0.84 ± 0.26  0.96 ± 0.27  0.91 ± 0.32  1.01 ± 0.35 | 73  73  72  71 | 0.91 ± 0.29  0.90 ± 0.26  0.86 ± 0.19  1.01 ± 0.29 | 25  25  25  25 | 0.91 ± 0.24  1.09 ± 0.27  0.97 ± 0.29  1.01 ± 0.35 | 26  26  26  26 |  | .578  .117  .623  1.000 | .613  .687  .739  .999 | .999  .058  .358  1.000 |

*Note*. SE_ap_ = Sample Entropy antero-posterior; PS = patients with psychomotor slowing; non-PS = patients without psychomotor slowing; HC = healthy controls; EO = eyes open, natural upright head position, and feet hip-width apart; EC = eyes closed natural upright head position, and feet hip-width apart; EOHR = eyes open, head reclined, and feet hip-width apart; ECHR = eyes closed, head reclined, and feet hip-width apart; N = Number of participants for the specific condition and group; sd = standard deviation; btw = between.

* p < 0.05

# ANOVA and Posthocs in postural parameters in Sway Path

**Methods**

We have also calculated the sway path from the raw data, for which group differences are shown in Table S23.

**Results**

PS were found to have a longer sway path in the antero-posterior direction and in total than HC. However, we found no group difference in the swaying distance in medio-lateral direction (Table S23).

Table S23. Effect of group and condition on sway path in medio-lateral, antero-posterior direction, and total.

|  | Main ANOVAs | | | |  |  | PS |  | non-PS |  | HC |  |  | p-value for posthocs btw groups | | |
| --- | --- | --- | --- | --- | --- | --- | --- | --- | --- | --- | --- | --- | --- | --- | --- | --- |
|  |  | F | numDF, denDF | p |  |  | mean ± sd | N | mean ± sd | N | mean ± sd | N |  | PS vs. HC | PS vs. non-PS | non-PS  vs HC |
| Sway path _medio-lateral_ | Group  Condition Group*Condition | 2.40  5.50  0.64 | 2,121  3,362  6,362 | .095  .001*  .701 | Conditions | EO  EC  EOHR  ECHR | 412.07 ± 141.88  431.75 ± 138.12  418.21 ± 151.63  444.99 ± 158.51 | 73  73  73  72 | 404.32 ± 123.15  408.70 ± 119.14  391.07 ± 119.35  407.25 ± 117.06 | 25  25  25  25 | 474.59 ± 140.88  485.24 ± 141.52  474.26 ± 130.42  488.00 ± 134.17 | 26  26  26  26 | Posthocs | .129  .221  .191  .383 | .969  .758  .682  .469 | .178  .130  .090  .104 |
| Sway path _antero-posterior_ | Group  Condition Group*Condition | 4.31  40.83  1.78 | 2,121  3,362  6,362 | .016*  <.001*  .102 |  | EO  EC  EOHR  ECHR | 458.01 ± 209.55  539.86 ± 198.19  476.85 ± 185.95  588.83 ± 212.63 | 73  73  73  72 | 498.93 ± 205.97  526.0622 ± 172.29  506.38 ± 174.87  575.28 ± 181.45 | 25  25  25  25 | 579.66 ± 218.20  630.65 ± 218.46  640.71 ± 275.64  715.09 ± 238.66 | 26  26  26  26 |  | .030  .136  .002*  .023* | .670  .955  .811  .957 | .347  .172  .057  .045* |
| Sway path _total_ | Group  Condition Group*Condition | 4.17  36.60  1.44 | 2,121  3,362  6,362 | .018*  <.001*  .199 |  | EO  EC  EOHR  ECHR | 679.08 ± 236.45  761.64 ± 223.48  701.01 ± 225.22  814.19 ± 255.02 | 73  73  73  72 | 705.61 ± 213.54  731.60 ± 185.39  702.04 ± 191.58  775.14 ± 194.92 | 25  25  25  25 | 814.85 ± 239.58  868.68 ± 233.68  868.95 ± 285.80  943.94 ± 265.73 | 26  26  26  26 |  | .032*  .113  .006*  .043* | .875  .843  .999  .746 | .219  .094  .031*  .029* |

*Note*. Sway path in medio-lateral or antero-posterior direction was calculated by summing the absolute distance between all values (formula: S(abs(x_j+1_ - x_j_))). Sway path_total_ was calculated using the raw CoP coordinates (x and y values) and calculating the Euclidean distance (formula: sqrt((x2 - x1)^2 + (y2 - y1)^2)) between all points.

PS = patients with psychomotor slowing; non-PS = patients without psychomotor slowing; HC = healthy controls; EO = eyes open, natural upright head position, and feet hip-width apart; EC = eyes closed natural upright head position, and feet hip-width apart; EOHR = eyes open, head reclined, and feet hip-width apart; ECHR = eyes closed, head reclined, and feet hip-width apart; N = Number of participants for the specific condition and group; sd = standard deviation; btw = between.

* p < 0.05

# ANOVA and Posthocs in postural parameters in Sway Velocity

**Methods**

We also calculated the sway velocity for which group differences are presented in Table S24.

**Results**

Regarding sway velocity, PS and HC differed in antero-posterior direction and in total for all conditions except EC. For EOHR and ECHR the sway velocity total also differed between non-PS and HC.

Table S24. Effect of group and condition on sway velocity in medio-lateral, antero-posterior direction, and total.

|  | Main ANOVAs | | | |  |  | PS |  | non-PS |  | HC |  |  | p-value for posthocs btw groups | | |
| --- | --- | --- | --- | --- | --- | --- | --- | --- | --- | --- | --- | --- | --- | --- | --- | --- |
|  |  | F | numDF, denDF | p |  |  | mean ± sd | N | mean ± sd | N | mean ± sd | N |  | PS vs. HC | PS vs. non-PS | non-PS  vs HC |
| Sway velocity _medio-lateral_ | Group  Condition Group*Condition | 3.13  10.74  1.08 | 2,122  3,355  6,355 | .047*  <.001*  .376 | Conditions | EO  EC  EOHR  ECHR | 0.79 ± 0.23  0.84 ± 0.24  0.79 ± 0.21  0.85 ± 0.24 | 70  72  70  70 | 0.80 ± 0.24  0.81 ± 0.24  0.78 ± 0.24  0.81 ± 0.23 | 25  25  24  25 | 0.93 ± 0.28  0.96 ± 0.28  0.95 ± 0.25  0.97 ± 0.26 | 27  27  27  27 | Posthocs | .059  .144  .039*  .132 | .999  .785  .903  .653 | .147  .095  .051  .058 |
| Sway velocity _antero-posterior_ | Group  Condition Group*Condition | 4.07  42.54  1.88 | 2,122  3,355  6,355 | 0.020*  <.001*  .084 |  | EO  EC  EOHR  ECHR | 0.87 ± 0.33  1.07 ± 0.40  0.95 ± 0.38  1.16 ± 0.43 | 70  72  70  70 | 0.99 ± 0.41  1.05 ± 0.34  0.96 ± 0.28  1.14 ± 0.36 | 25  25  24  25 | 1.14 ± 0.43  1.24 ± 0.43  1.25 ± 0.55  1.40 ± 0.48 | 27  27  27  27 |  | .023*  .174  .003*  .029* | .538  .952  .834  .974 | .410  .205  .072  .061 |
| Sway velocity  _total_ | Group  Condition Group*Condition | 4.49  42.87  2.03 | 2,122  3,355  6,355 | .013*  <.001*  .061 |  | EO  EC  EOHR  ECHR | 1.29 ± 0.37  1.50 ± 0.44  1.36 ± 0.41  1.59 ± 0.47 | 70  72  70  70 | 1.40 ± 0.42  1.45 ± 0.37  1.36 ± 0.34  1.54 ± 0.39 | 25  25  24  25 | 1.60 ± 0.48  1.71 ± 0.47  1.71 ± 0.56  1.86 ± 0.53 | 27  27  27  27 |  | .019*  .114  .003*  .025* | .737  .860  .976  .862 | .249  .100  .029*  .029* |

*Note*. Sway velocity in medio-lateral or antero-posterior direction was calculated by summing the absolute distance between all values (formula: S(sqrt(x_j+1_ - x_j_)^2)) divided by 1000 and multiplied by 2 to get the distance per minute [m/min]. Sway velocity_total_ was calculated using the raw CoP coordinates (x and y values) and calculating the Euclidean distance (formula: sqrt((x2 - x1)^2 + (y2 - y1)^2)) divided by 1000 and multiplied by 2 between all points.

PS = patients with psychomotor slowing; non-PS = patients without psychomotor slowing; HC = healthy controls; EO = eyes open, natural upright head position, and feet hip-width apart; EC = eyes closed natural upright head position, and feet hip-width apart; EOHR = eyes open, head reclined, and feet hip-width apart; ECHR = eyes closed, head reclined, and feet hip-width apart; N = Number of participants for the specific condition and group; sd = standard deviation; btw = between.

* p < 0.05

# Abbreviations

| PS | patients with psychomotor slowing |
| --- | --- |
| non-PS | patients without psychomotor slowing |
| HC | healthy controls |
| RMS | Root Mean Square |
| CI | Complexity Index |
| ml | medio-lateral |
| ap | antero-posterior |
| EO | eyes open, head in a neutral position |
| EC | eyes closed, head in a neutral position |
| EOHR | eyes open, head reclined |
| ECHR | eyes closed, head reclined |
| EOTS | eyes open, head in a neutral position, tandem stand |
| ECTS | eyes closed, head in a neutral position, tandem stand |
| OCoPS-P | Overcoming Psychomotor Slowing in Psychosis |
| CoP | Centre of Pressure |
| PANSS | Positive And Negative Symptom Scale |
| SRRS | Salpêtrière Retardation Rating Scale |
| mSRRS | Brief Negative Symptom Scale |
| BFCRS | Bush-Francis Catatonia Rating Scale |
| NES | Neurological Evaluation Scale |
| UPDRS | Unified Parkinson Disease Rating Scale |
| BNSS | Brief Negative Symptom Scale |
| OLZ eq. | olanzapine equivalents |
| BMI | Body Mass Index |
| NSS | Neurological Soft Signs |
| CCTCC | cortico-cerebellar-thalamic-cortical circuit |
